# True Randomness from Big Data

**DOI:** 10.1038/srep33740

**Published:** 2016-09-26

**Authors:** Periklis A. Papakonstantinou, David P. Woodruff, Guang Yang

**Affiliations:** 1 Rutgers University, MSIS, Piscataway, NJ 08853, USA; 2 IBM Research Almaden, San Jose, CA 95120, USA; 3 Institute for Computing Technology, CAS, Beijing, 100190, China

## Abstract

Generating random bits is a difficult task, which is important for physical systems simulation, cryptography, and many applications that rely on high-quality random bits. Our contribution is to show how to generate provably random bits from uncertain events whose outcomes are routinely recorded in the form of *massive data sets*. These include scientific data sets, such as in astronomics, genomics, as well as data produced by individuals, such as internet search logs, sensor networks, and social network feeds. We view the generation of such data as the sampling process from a *big source*, which is a random variable of size at least a few gigabytes. Our view initiates the study of big sources in the randomness extraction literature. Previous approaches for big sources rely on statistical assumptions about the samples. We introduce a general method that provably extracts almost-uniform random bits from big sources and extensively validate it empirically on real data sets. The experimental findings indicate that our method is efficient enough to handle large enough sources, while previous extractor constructions are not efficient enough to be practical. Quality-wise, our method at least matches quantum randomness expanders and classical world empirical extractors as measured by standardized tests.

Randomness extraction is a fundamental primitive, and there is a large body of work on this; we refer the reader to the recent surveys^1^,^2^ and references therein. The extracted true random bits are critical for numerical simulations of non-linear and dynamical systems, and also find a wide array of applications in engineering and science^3^,^4^,^5^,^6^,^7^. There are tangible benefits to linking randomness extraction to big sources. First, big sources are now commonplace^8^,^9^. Second, since they are in common use, adversaries cannot significantly reduce statistical entropy without making them unusable^10^. In addition, the ability to extract from big samples leverages the study of classical-world extractors^11^ and quantum randomness expanders^12^, since they allow us to post-process while ignoring local statistical dependencies. The contribution of our work is to give an extractor which has theoretical guarantees and which works efficiently, in theory and in practice, on massive data sets. In particular the model of processing massive data we study is the data stream model discussed in more detail below.

The main obstruction in big source extraction is the lack of available computational resources. Previously, the study of general extractors was largely theoretical. Note that no known theoretical construction could be applied even on samples of modest size, e.g., 10 megabytes (MB). Even if it had been possible to gracefully scale the performance of previous extractors, processing a 20 gigabyte (GB) sample would have taken more than 100,000 years of computation time and exabytes of memory. In contrast, our proposed method processes the same sample using 11 hours and 22 MB of memory. The proposed method is the first feasibility result for big source extraction, but is also the first method that works in practice.

## Extractors from Big Sources

Let us now state things more precisely. Randomness can be extracted from an (*n*, *k*)-source *X*, where *X* is a random variable over {0, 1}^*n*^ whose *min-entropy* 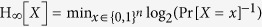 is at least *k*. The *min-entropy rate* of *X* is *κ* = H_∞_[*X*]/*n*. Min-entropy is a worst-case statistic and in general cannot be replaced by the average-case *(Shannon) entropy* 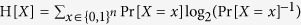, an issue that we will elaborate more on later. To extract randomness from a source *X*, we need: (i) a sample from *X*, (ii) a small uniform random seed *Y*, (iii) a lower bound *k* for H_∞_[*X*], and (iv) a fixed error tolerance *ε* > 0. Formally, a (*k*, *ε*)*-extractor* Ext: {0, 1}^*n*^ × {0, 1}^*d*^ → {0, 1}^*m*^ outputs Ext (*X*, *Y*) that is *ε-close to the uniform distribution*, i.e., 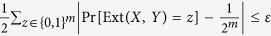, when taking input from any (*n*, *k*)-source *X* together with a random seed *Y*. In typical settings 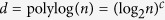 for a constant *c* > 0, and *m* > *d*. The seed is necessary since otherwise it is impossible to extract a single random bit from one source^2^. We note that other notions of the output being random, other than closeness to the uniform distribution, are possible and have been studied in a number of general science journal articles^13^,^14^,^15^,^16^. These are based on measures of randomness such as approximate entropy. Since our measure is *total variation distance* to the uniform distribution, our generated output provably appears random to *every* other specific measure, including e.g., approximate entropy.

What does it mean to extract randomness from big sources? Computation over big data is commonly formalized through the *multi-stream model of computation*, where, in practice, each stream corresponds to a hard disk^17^. Algorithms in this model can be turned into executable programs that can process large inputs. Formally, a *streaming extractor* uses a local memory and a constant number (e.g., two) of streams, which are *tapes* the algorithm reads and writes sequentially from left to right. Initially, the sample from *X* is written on the first stream. The seed of length *d* = polylog(*n*) resides permanently in local memory. In each computation step, the extractor operates on one stream by writing over the current bit, then moving on to the next bit, and finally updating its local memory, while staying put on all other streams. The sum *p* of all passes over all streams is constant or slightly above constant and the local memory size is *s* = polylog(*n*).

The limitations of streaming processing (tiny *p* and *s*) pose challenges for randomness extraction. For example, a big source *X* could be controlled by an adversary who outputs *n*-bit samples *x* = *x*_1_*x*_2_…*x*_*n*_ where 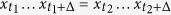, for some *t*_1_ ≠ *t*_2_ and large integer Δ > 0. Besides such simple dependencies, an extractor must eliminate all possible determinacies without knowing any of the specifics of *X*. To do that, it should spread the input information over the output, a task fundamentally limited in streaming algorithms. This idea was previously^18^ formalized, where it was shown that an extractor with only one stream needs either polynomial in *n*, denoted poly(*n*), many passes, or poly(*n*)-size local memory; i.e., *no single-stream extractor exists*. Even if we add a constant number of streams to the model, the so-far known extractors^19^,^20^ cannot be realized with 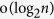 many passes (a corollary^17^), nor do they have a known implementation with tractable stream size.

An effective study on the limitations of every possible streaming extractor goes hand in hand with a concrete construction we provide. The main purpose of this article is to explain why such a construction is at all possible and our focus here is on the empirical findings. The following theorem relies on mathematical techniques that could be of independent interest (Supplementary Information pp. 26–30) and states that 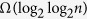 many passes are necessary for all multi-stream extractors. This constitutes our main impossibility result. This unusual, slightly-above-constant number of passes, is also sufficient, as witnessed by the two-stream extractor presented below.

**Theorem.** *Fix an arbitrary multi-stream extractor* Ext: {0, 1}^*n*^ × {0, 1}^*d*^ → {0, 1}^*m*^ *with error tolerance ε* = 1/poly(*n*), *such that for every input source X where* H_∞_[*X*] ≥ *κn*, *for any constant κ* > 0, *and uniform random seed Y, the output* Ext (*X*, *Y*) *is ε*-*close to uniform. If Ext uses sub-polynomial n*^*o*(1)^ *local memory then it must make* 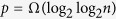 *passes. Furthermore, the same holds for every constant* 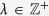 *number of input sources*.

## Our RRB Extractor

**Figure 1:**
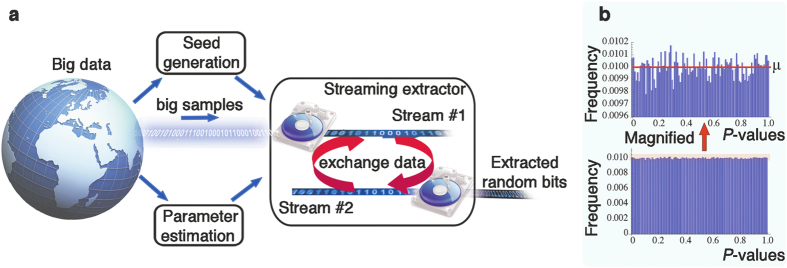
High-level overview of the extraction method. (**a**) The core of the proposed method is the streaming extractor. The description corresponds to one big source, with parameter estimation and initial seed generation done only once. Repeating the seed generation is optional, and is not needed for practical purposes. (**b**) The output of the proposed method is validated by standard statistical tests (NIST). On the ideal (theoretical) uniform distribution the P-values are uniformly distributed. We plot the histogram for 1,512,000 P-values proving a high-level indication about the uniformly extracted bits, i.e., well-concentrated frequencies around the expected frequency *μ* = 0.01. This graph provides a visual overview (averaging), whereas detailed statistics are in the Supplementary Information.

We propose and validate a new empirical method for true randomness extraction from big sources. This method consists of a novel extractor and empirical methods to both estimate the min-entropy and generate the initial random seed. Figure 1 depicts a high-level view of the complete extaction method. This is the first complete general extraction method, not only for big sources but for every statistical source.

We propose what we call the *Random Re-Bucketing* (RRB) extractor. For our RRB extractor we prove (Supplementary Information pp. 11–25) that it outputs almost-uniform random bits when given as input a single sample from an arbitrary weak source – as long as the source has enough min-entropy. Mathematical guarantees are indispensable for extractors, since testing uniformity and estimating entropy of an unknown distribution, even approximately, is computationally intractable^21^.

**Figure 2:**
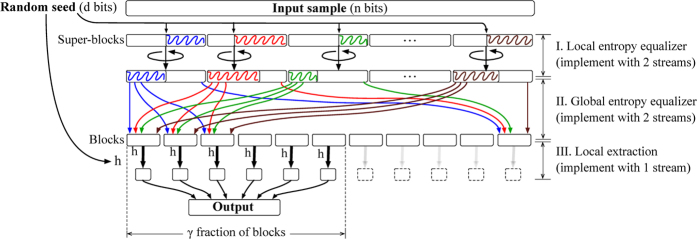
The Random Re-Bucketing (RRB) extractor. The random seed of size polylog(*n*) is used only in Stages I & III. In Stage III the same local extractor *h* is used for the first *γ* fraction of blocks. The number of super-blocks *b* also depends on an error tolerance *ε* and the empirically estimated min-entropy rate *κ*. In the main body, we explain how to realize this description as an algorithm that uses *two streams*.

**Figure 3:**
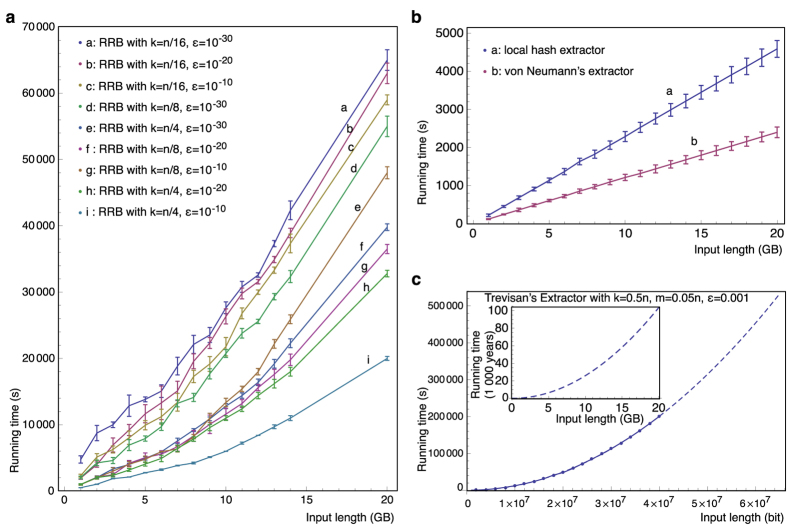
Running time of RRB compared with other extractors. Running time is measured on input samples of size 1 GB–20 GB for von Neumann extractor, local hash extractor (with block-size 1024 bits), and for RRB (with *k* = *n*/4, *n*/8, *n*/16 and *ε* = 10^−10^, 10^−20^, 10^−30^). Trevisan’s extractor is only measured for *ε* = 0.001 on samples of size up to 5 MB = 4 × 10^7^ bits, since the available implementations of finite fields cannot handle larger samples or smaller *ε*. The running time of Trevisan’s extractor on larger input size (in particular, 103,407 years for 20 GB input) is estimated by polynomial fitting assuming all data in the main memory, which is an unrealistic advantage. The exact form of the fittest polynomial is determined through cross-validation and standard analysis of polynomial norms.

A key-feature of the RRB extractor in Fig. 2 is its simplicity, with the technical difficulty being in proving its correctness, which requires a novel, non-trivial analysis. RRB is the first extractor capable of handling big sources without additional assumptions. Previous works require either (i) unrealistic running times or (ii) ad hoc assumptions about the source. In particular, the local extractors such as von Neumann^22^ and Local Hash fail significantly in terms of output quality, whereas the throughput of Trevisan’s extractor^19^ and its followups degrade significantly (see Fig. 3) with the size of the sample^12^ even with practical optimization considered; e.g., 103,407 years of computing time for a 20 GB input sample and *ε* = 10^−3^, *κ* = 1/2. We note that we choose to compare to the Local Hash and von Neumann extractors since these are the only extractors experimented upon in previous work (see ref. 23 for empirical work using von Neumann’s extractor, and see refs 12, 24 and 25 for empirical work using Local Hash), and importantly, both extractors happen to be streaming extractors. Thus, due to their special attention in previous work they are two ideal candidates for comparison. We refer the reader to Table 2, Fig. 3, and the Supplementary Information for details.

The RRB extractor consists of the following three stages.

-  Partition the *n*-bit long input into 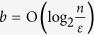 many *super-blocks*, each of length *n*/*b*. Inside each super-block, choose uniformly and independently a random point to cyclically shift the super-block.
-  Re-bucket the *b super-blocks* into *n*/*b* many *blocks* each of size *b*, where the *i*-th block consists of the *i*-th bit from every (shifted) super-block, for *i* = 1, 2, …, *n*/*b*.
-  Specify a *local extractor h*: {0, 1}^*b*^ → {0, 1}^*κb*/2^ using the uniform random seed; for example, *h* can be a random Toeplitz matrix. Then, locally apply *h* on the first *b*_*O*_ = *γn*/*b* blocks, concatenate, and output the result. Here the *effectiveness factor γ* = Ω(1) denotes the fraction of blocks used for local extraction.

This extractor can be realized with two streams and local memory size polylog(*n*) (more details of streaming realization on p. 5). (I) Cyclically shift every super-block using in total 4 passes (every pass operates on all super-blocks). (II) Re-bucket with 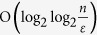 many passes and iterations where, in each iteration, the first and the second half of the first stream are shuffled with the help of the second stream. (III) Locally extract with 2 passes. The implementation is scalable since it uses 
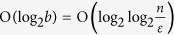
 passes and a 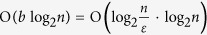 bit random seed. For example, 44 passes and 57 KB of a random seed suffice to extract 1 GB of randomness from a 20 GB input. (For min-entropy *k* ≥ 0.2*n* = 4 GB, and error rate *ε* = 10^−27^ < 1/*n*^2^. With a total of 50 passes, the error rate can be as small as *ε* = 10^−100^. Most of the seed is used to sample a random Toeplitz hash *h*.).

Stage III with *γ* = 1 has been used before^26^,^27^,^28^ in randomness extraction from sources of guaranteed *next-block-min-entropy*. This guarantee means that *every* block, as a random variable, has enough min-entropy left even after revealing all the blocks preceding it, i.e., it presumes strong inter-block independence. Such a precondition restricts the applicability of Stage III since it appears too strong for common big sources, especially when there is an adversary. However, by introducing Stages I & II we can provably fulfill the precondition for a theoretically lower bounded constant *γ*. In practice, a larger (i.e., better than in theory) *γ* can be empirically found and validated.

Stage I equalizes entropy within each super-block and, subsequently, Stage II distributes entropy globally. After Stage II, the following property holds. Let 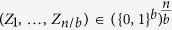 denote the blocks of bits of the intermediate result at the end of Stage II. *Next-block-min-entropy* H_∞_[*Z*_*i*_|*Z*_*i*−1_, …, *Z*_1_] 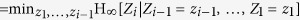 is the min-entropy of the *i*-th block *Z*_*i*_ conditioned on the worst choice of all the blocks preceding *Z*_*i*_. We show (Supplementary Information pp. 15–25) that *for every i* ∈ {1, …, *b*_*O*_},


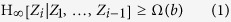


with probability (over the choice of the random seed) greater than 1 − *ε*/*n*, for 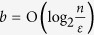, *γ* = Ω(1) and *b*_*O*_ = Ω(*n*/*b*). Therefore, Stage III extracts *ε*-close to uniform random bits.

To invoke the extractor, it is necessary to find an initial random seed and estimate the min-entropy rate *κ* of the source. The proposed method includes an empirical realization of a multi-source extractor to obtain 4 MB initial randomness from 144 audio samples each of 4 MB. We also propose and validate an empirical protocol that estimates both *κ* and *γ* simultaneously by combining RRB itself with standardized uniformity tests.

Finally, we note that the RRB extractor bears some superficial similarities to the Advanced Encryption Standard (AES) block cipher, which is an encryption scheme widely used in practice. That is, at a high level both schemes efficiently mix information, though they do so in very different ways, e.g., in AES this is done on a much more local scale, whereas we mix information globally. Moreover, unlike the RRB extractor that we propose, the AES block cipher cannot have provable guarantees without proving that *P* ≠ *NP*.

## Methods

The proposed method is validated in terms of efficiency and quality, measured by standard quality test suites, NIST^29^ and DIEHARD^30^. The results strongly support our new extractor construction on real-world samples. The empirical study compares multiple extraction methods on many real world data sets, and demonstrates that our extractor is the only one that works in practice on sufficiently large sources.

Our experiments are explained in more detail below and we summarize them here. Our samples range in size from 1.5 GB–20 GB and they are from 12 data categories: compressed/uncompressed text, video, images, audio, DNA sequenced data, and social network data. The empirical extraction is for *ε* = 10^−20^ and estimated min-entropy rate ranging from 1/64 to 1/2, with extraction time from 0.85 hours to 11.06 hours on a desktop PC (Fig. 3). The extracted outputs of our method pass all quality tests, whereas the before-extraction-datasets fail almost everywhere (Tables 1 and 2). The output quality of RRB is statistically identical to the uniform distribution. Such test results provide further evidence supporting that the extraction quality is close to the ideal uniform distribution, besides the necessary^31^ rigorous mathematical treatment.

### Extraction method

The complete empirical method consists of: (i) initial randomness generation, (ii) parameter estimation, and (iii) streaming extraction. Components (ii) and (iii) rely on initial randomness.

We first extract randomness from multiple independent sources without using any seed. Then, we use RRB to expand this initial randomness further.

Parameter estimation determines a suitable pair (*κ*, *γ*) of min-entropy rate *κ* = *k*/*n* and effectiveness factor 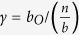.

### Experimental set-up

We empirically evaluate the quality and the efficiency of our RRB extractor.

Quality evaluation is performed on big samples from twelve semantic data categories: compressed/uncompressed audio, video, images, text, DNA sequenced data, and social network data (for audio, video, and images the compression is lossy). The initial randomness used in our experiments consists of 9.375 × 10^8^ bits ≈117 MB generated from 144 pieces of 4 MB compressed audio and one piece of 15 GB compressed video. The produced randomness is used for parameter estimation on samples ranging in size from 1 GB to 16 GB from each of the 12 categories. The estimated *κ* and *γ* vary within [1/64, 1/2] and [1/32, 1/2] respectively, cross-validated (i.e., excluding previously used samples) on samples of size 1.5 GB–20 GB with error tolerance *ε* = 10^−20^. Final extraction quality is measured on all 12 categories by the standard NIST and DIEHARD batteries of statistical tests.

Operating system kernel-level measurements are taken for the running time and memory usage of RRB. These measurements are taken from RRB on input sizes 1 GB–20 GB, min-entropy rate *κ* ∈ {1/4, 1/8}, and error tolerance *ε* ∈ {10^−10^, 10^−20^}.

For comparison, we measure quality and efficiency for three of the most popular representatives of extractors. The quality of Local Hash and von Neumann extractors is evaluated on 12 GB of raw data (from the 12 categories) and on 12 GB adversarial synthetic data. The efficiency is measured for von the Neumann extractor, Local Hash, and Trevisan’s extractor. See the Supplementary Information for tables and figures showing this.

### Empirical initial randomness generation

Seeded extraction, as in RRB, needs uniform random bits to start. All the randomness for the seeds in our experiments is obtained by the following method (which we call it *randomness bootstrapping*) in two phases: (i) obtain initial randomness *ρ* through (seedless) multiple-independent-source extraction, and (ii) use *ρ* for parameter estimation and run RRB to extract a longer string *ρ*_*long*_, 
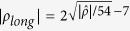
, where 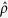 is the part of *ρ* used as the seed of RRB during bootstrapping. By elementary information theory, *ρ*_*long*_ can be used instead of a uniformly random string.

Phase (i) is not known to have a streaming implementation, which is a not an issue since it only extracts from small samples. Start with 144 statistically independent compressed audio samples *ρ*_1_, …, *ρ*_144_: each sample is 4 MB of high-quality (320 Kbps) compressed recording (MPEG2-layer3). Taken together, the samples last 4.1 hours. These samples are generated privately – without malicious adversarial control – using different independent sound-settings and sources. Partition the samples into 16 groups, each consisting of 9 = 3^2^ samples. Every *ρ*_*i*_ can be interpreted as a field element in GF[*p*], where *p* = 2^57885161^ − 1 is the largest known Mersenne prime and 4 MB 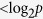 bits. For the first group {*ρ*_1_, …, *ρ*_9_}, compute *ρ*^(1)^ = *ρ*′ ⋅ *ρ*′′ + *ρ*′′′ where *ρ*′ = *ρ*_1_ ⋅ *ρ*_2_ + *ρ*_3_, *ρ*′′ = *ρ*_4_ ⋅ *ρ*_5_ + *ρ*_6_, and *ρ*′′′ = *ρ*_7_ ⋅ *ρ*_8_ + *ρ*_9_; which is a two-level recursion. In the same way, compute *ρ*^(2)^, …, *ρ*^(16)^ and finally let *ρ* = *ρ*^(1)^ + … + *ρ*^(16)^, with all operations in GF[*p*]. We call this the BIWZ method due to the authors^32^,^33^ who studied provable multi-source extraction based on the field operation *x* ⋅ *y* + *z*.

Phase (ii) uses the 4 MB extracted by BIWZ out of which 3.99 MB are used in parameter estimation for compressed video. The remaining 10 KB are used to run RRB on 15 GB compressed video, which is generated and compressed privately, i.e., without adversarial control. Our hypothesis is that the estimated parameters are valid for RRB, i.e., *n* bits of compressed video contain min-entropy *n*/2 that can be extracted by RRB with effectiveness factor *γ* = 1/32. This hypothesis is verified experimentally. With the given seed and *κ* = 1/2, *γ* = 1/32, and *ε* = 10^−100^, RRB extracts the final 9.375 × 10^8^ random bits.

### Empirical parameter estimation protocol

There are two crucial parameters for RRB: the min-entropy rate *κ* and the effectiveness factor *γ*. In theory, *γ* is determined by *κ*, *n*, *ε*. In practice, better, empirically validated values are estimated simultaneously for *κ* and *γ*. This works because in addition to min-entropy, *κ* induces the next-block-min-entropy guarantee for a fraction of *γ* blocks.

For every semantic data category, the following protocol estimates a pair of (*κ*, *γ*).

First, obtain a bit sequence *s* of size 1 GB by concatenating sampled < 1 MB segments from the target data category. Then, compress *s* into *s*′ using LZ77^34^ (*s*′ = *s* if *s* is already compressed). Since the ideal compression has |*s*′| equal to the Shannon entropy of *s*, the compression rate 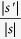 is also an upper bound for the min-entropy rate. To obtain a lower bound for the min-entropy rate (required parameter for RRB), we start from 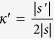 and search inside [0, *κ*′]. For min-entropy rate 
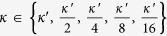
 and effectiveness factor 
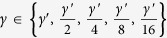
, extract from *s* using RRB, with parameters *κ*, *γ*, and *ε* = 10^−20^ and seed from the initial randomness. Apply NIST tests on the extracted bits for every (*κ*, *γ*) pair. If the amount of extracted bits is insufficient for NIST tests, then start over with an *s* twice as long. We call a pair of (*κ*_0_, *γ*_0_) *acceptable* if NIST fails with frequency at most 0.25% for every run of RRB with parameters *κ* ≤ *κ*_0_ and *γ* ≤ *γ*_0_. This 0.25% threshold is conservatively set slightly below the expected failure probability of NIST on ideal random inputs, which is 0.27%. If (*κ*_0_, *γ*_0_) is a correctly estimated lower bound, then every estimate (*κ*, *γ*) with *κ* ≤ *κ*_0_ and *γ* ≤ *γ*_0_ is also a correct lower bound. Hence, the extraction with (*κ*, *γ*) should be random and pass the NIST tests. We choose the acceptable pair (if any) that maximizes the output length.

There is strong intuition in support of the correct operation of this protocol. First, the random sampling for *s* preserves with high probability the min-entropy rate^35^. Second, an extractor cannot extract almost-uniform randomness if the source has min-entropy much lower than the estimated one. Finally, NIST tests exhibit a certain ability to detect non-uniformity. Verification of the estimated parameters is done by cross-validation.

### Streaming realization of the RRB extractor

The streaming extractor uses 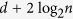 bits local memory and 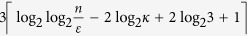 passes over two streams, for input length *n*, min-entropy rate *κ*, error tolerance *ε* and seed length *d*. RRB is also parametrized by the effectiveness factor *γ* as shown below.

Given *n*, *ε*, and the estimated *κ*, *γ*, we initially set *k* = *κn*, the output length 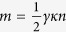, and the number of super-blocks 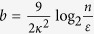. For convenience, *n* is padded to a power of 2, *κ* and *γ* are rounded down to an inverse power of 2, and *b* is rounded up to a power of 2. Hereafter, no further rounding is needed. Let *σ*_1_ and *σ*_2_ denote two read/write streams. The input sample *x* ∈ {0, 1}^*n*^ is initially on *σ*_1_. Obtain a seed *y* of length 
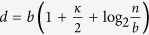
 from the initially generated randomness, and store it in local memory. We interpret *y* as 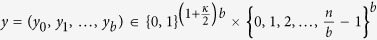.

In Stage I, we partition the input into *b* super-blocks 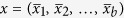, where 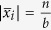 for every 
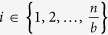
. RRB reads *x* from *σ*_1_ and writes 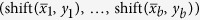 to *σ*_2_, where every 
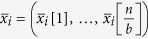
, 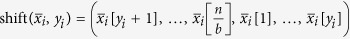 denotes the cyclic shift of 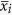 with offset *y*_*i*_. This can be done with 4 passes.

In Stage II, we compute the re-bucketing of 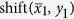, …, 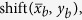 which is stored on *σ*_2_. The re-bucketing output is denoted by (*z*_1_, …, *z*_*n*/*b*_), where every *z*_*j*_ collects the *j*-th bit from all shifted super-blocks, i.e., 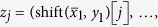 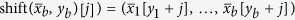. The re-bucketing of *b* super-blocks can be done with 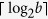 iterations, where every iteration reduces the number of super-blocks by a factor of two by interlacing (with the help of *σ*_1_) the first and second half of *σ*_2_. In particular, the first iteration merges every pair of 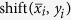 and 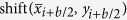 into a single super-block 
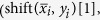
 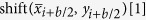, …, 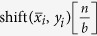, 
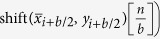
, which consists of *n*/*b* blocks (i.e. 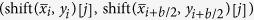 for *j* = 1, 2, …, *n*/*b*) each of length 2. During the 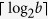 many iterations, RRB spends 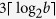 passes to compute (*z*_1_, …, *z*_*n*/*b*_) and store it on *σ*_1_.

In the final stage, we output 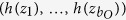 to *σ*_2_, where *h*: {0, 1}^*b*^ → {0, 1}^*κb*/2^ is a hash function realized through a Toeplitz matrix specified by *y*_0_ from the seed and *b*_*O*_ = *γn*/*b* the number of blocks used for the output. This *m*-bit-long output can be locally extracted with 2 passes.

Therefore, RRB extracts *m* bits with 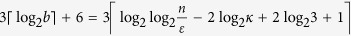 passes. The local memory size is dominated by Stage I, which requires 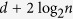 bits to store the seed and two counters for head positions.

The above description is for the estimated (*κ*, *γ*). If there is theoretical knowledge for *κ* and the error tolerance *ε* is given, then RRB provably extracts *m* = Ω(*n*) bits that are *ε*-close to uniform with 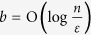 and *γ* = Ω(1). For instance, RRB provably works for 
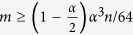
 and 
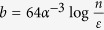
, *γ* = *α*/4, where *α* = *κ*^2^/(6 log*κ*^−1^) is a constant.

### Empirical statistical tests

Each statistical test measures one property of the uniform distribution by computing a P-value, which on ideal random inputs is uniformly distributed in [0, 1]. For each NIST test, subsequences are derived from the input sequence and P-values are computed for each subsequence. A significance level ***α*** ∈ [0.0001, 0.01] is chosen such that a subsequence passes the test whenever P-value ≥ ***α*** and fails otherwise. If we think that NIST is testing ideal random inputs, then the proportion of passing subsequences has expectation 1 − ***α***, and the *acceptable range of proportions* is the confidence interval chosen within 3 standard deviations. Furthermore, a second-order P-value is calculated on the P-values of all subsequences via a *χ*^2^-test. An input passes one NIST test if (i) the input induces an acceptable proportion and (ii) the second-order P-value ≥ 0.0001. An input passes one DIEHARD-test if P-value is in [***α***, 1 − ***α***].

We compare the statistical behavior of bits produced by our method with ideal random bits. For ideal random bit-sequences, ***α*** is the *ideal failure rate*. Anything significantly lower or higher than this indicates non-uniform input. In our tests, we choose the largest suggested significance level ***α*** = 0.01; i.e., the hardest to pass the test. All tests on our extracted bits appear statistically identical to ideal randomness. See the Supplementary Information for details.

### Experimental platform details

The performance of the streaming RRB, von Neumann extractor, and Local Hash is measured on a desktop PC, with Intel Core i5 3.2 GHz CPU, 8 GB RAM, two 1 terabyte (TB) hard drives and kernel version Darwin 14.0.0. The performance of Trevisan’s extractor is measured on the same PC with the entire input and intermediate results stored in main memory. We use the following software platforms and libraries. TPIE^36^ is the C++ library on top of which we implement all streaming algorithms – TPIE provides application-level streaming I/O interface to hard disks. For arbitrary precision integer and Galois field arithmetic we use GMP^37^ and FGFAL^38^. Mathematica^39^ is used for data processing, polynomial fitting, and plots. Source code is available upon request.

### Conclusion

We introduced the study of big source extraction, proposed a novel method for achieving this, and demonstrated its feasibility in theory and practice. Big source extraction has immediate gains and poses new challenges, while opening directions in the intersection of randomness extraction, data stream computation, mathematics of computation and statistics, and quantum information. We refer the reader to the Supplementary Information for details of proofs and experiments.

## Additional Information

**How to cite this article**: Papakonstantinou, P. A. *et al.* True Randomness from Big Data. *Sci. Rep.* **6**, 33740; doi: 10.1038/srep33740 (2016).

## Supplementary Material

Supplementary Information

## Figures and Tables

**Table 1:** Empirically extracted bits versus ideal (theoretical) uniform random bits.

Data category	NIST Test Suite		DIEHARD Test Suite	
	# of tests	Observed(Ideal) freq.	# of tests	Observed(Ideal) freq.
Compressed audio	564	0 (2)	144	2 (3)
Comp. video	752	4 (2)	144	4 (3)
Comp. images	752	1 (2)	144	2 (3)
Comp. social network data	3008	9 (8)	576	10 (12)
Comp. DNA sequenced data	752	1 (2)	144	3 (3)
Comp. text	3008	11 (8)	576	14 (12)
Audio	752	2 (2)	144	1 (3)
Video	752	1 (2)	144	1 (3)
Images	564	0 (2)	62	2 (1)
Social network data	2256	6 (6)	432	5 (9)
DNA sequenced data	752	2 (2)	144	2 (3)
Text	1504	1 (4)	288	7 (6)
**Total**	15416	38 (45)	2942	53 (61)

Overall statistics of the empirically extracted bits using the proposed method. The second column is the total number of NIST tests per data category. The third column compares the number of NIST tests that the empirically extracted bits do not pass with the expected number of NIST tests (listed in parenthesis) that the ideal uniform random bits will not pass. Any number significantly above or below the one in parenthesis indicates non-uniformity (discussed in Empirical statistical tests on p. 5). Similarly, the fourth and fifth columns report the results for DIEHARD tests. Further statistics related to this table are reported in Supplementary Information Table S1.

**Table 2:** Comparative extraction quality performance.

Extractor and data setting	NIST Test Suite		
	Number of tests	Observed (Ideal) freq.	P-val < 0.0001
Raw data	2256	1931 (6.09)	1561 (0.22)
Von Neumann	2256	966 (6.09)	785 (0.22)
Von Neumann on adversary	2256	1507 (6.09)	1435 (0.22)
Local hash	2256	16 (6.09)	1 (0.22)
Local hash on adversary	2256	781 (6.09)	170 (0.22)
RRB	2256	4 (6.09)	0 (0.22)
RRB on adversary	2256	5 (6.09)	1 (0.22)

The raw data consists of 12 files each of size 1000 MB from the 12 data categories and the adversarial data are generated by simply replacing 10 MB in each file with fixed values. NIST tests are applied on the raw data and extraction output of von Neumann, local hash, and RRB extractors on raw data and adversarial data. The second column is the total number of NIST tests per setting. The third column is the number of NIST tests that fail because of proportion, and the fourth column is the number of NIST tests that fail because of the second-order P-value. All are compared with the expected number of the ideal uniform random bits. Except from RRB and “RRB on adversary”, all other test results indicate non-uniform output (i.e. noticeably different from ideal uniform).
