# Supplementary material for: True Randomness from Big Data
Source: Sci Rep. 2016 Sep 26;6:33740. doi: 10.1038/srep33740 (PMC5036032; doi:10.1038/srep33740)
Supplement: Supplementary Information [file srep33740-s1.pdf]

Supplementary Information

— Methods, experimental results, and mathematical analysis

True randomness from big data
by Periklis A. Papakonstantinou, David Woodruff, and Guang Yang

Contents

1 Experimental data **1**

2 Mathematical analysis **6**
2.1 Summary of theoretical results . . . 6
2.2 Theoretical background and our techniques . . . 6
2.3 Notation and terminology . . . 8
2.4 The random re-bucketting (RRB) extractor . . . 10
2.4.1 Streaming extraction from bit-fixing sources . . . 11
2.5 Streaming extraction from general weak sources with RRB extractor . . . 14
2.5.1 Proof intuition of Theorem 8 . . . 14
2.5.2 A useful definition of goodness and important lemmas . . . 15
2.5.3 The RRB next-block-min-entropy guarantee . . . 20
2.5.4 Proof of Lemma 17 – from min-entropy to the good block guarantee . . . 21
2.5.5 Proof of Lemma 18 – from good blocks to next-block-min-entropy . . . 23
2.6 Lower bounds for streaming extractors . . . 25
2.6.1 Dependency graphs and dependency trees . . . 25
2.6.2 Proof of Theorem 21 – the general lower bound for randomness extractors . . . 26
2.6.3 Proof of Theorem 23 – the limitation of oblivious extractors . . . 28

1 Experimental data

Table S1: **Details on empirically extracted bits versus theoretical (ideal uniform) random bits.**

Data category	NIST Test Suite			DIEHARD Test Suite	
	**Number of tests**	**Observed (Ideal) freq.**	$\mathcal{P}$**-val** < 0.0001	**Number of tests**	$\mathcal{P}$**-val** < 0.01 **or** $\mathcal{P}$**-val** > 0.99
Compressed audio	564	0 (1.52)	0 (0.06)	144	2 (2.88)
Compressed video	752	4 (2.03)	0 (0.08)	144	4 (2.88)
Compressed images	752	1 (2.03)	0 (0.08)	144	2 (2.88)
Compressed social network data	3008	9 (8.12)	0 (0.30)	576	10 (11.52)
Compressed DNA sequenced data	752	1 (2.03)	0 (0.08)	144	3 (2.88)
Compressed text	3008	11 (8.12)	0 (0.30)	576	14 (11.52)
Uncompressed audio	752	2 (2.03)	0 (0.08)	144	1 (2.88)
Uncompressed video	752	1 (2.03)	0 (0.08)	144	1 (2.88)
Uncompressed images	564	0 (1.52)	0 (0.06)	62	2 (1.24)
Uncompressed social network data	2256	5 (6.09)	1 (0.23)	432	5 (8.64)
Uncompressed DNA sequenced data	752	2 (2.03)	0 (0.08)	144	2 (2.88)
Uncompressed text	1504	0 (4.06)	1 (0.15)	288	7 (5.76)
**Total**	15416	36 (41.62)	2 (1.62)	2942	53 (58.84)

The first column is the tested data categories. The second column is the total number of NIST tests per data category. The third column is the number of NIST tests with a passing proportion outside the confidence interval (see the section of "Empirical statistical tests" on page 4 of the main manuscript). The fourth column is the number of NIST tests that the second-order $\mathcal{P}$-value is less than 0.0001. Both of them are compared with the expected statistic of NIST tests on ideal uniform random bits, which is listed in parenthesis. Similarly, the fifth column is the total number of DIEHARD tests and the sixth the number of failing tests (in parenthesis is the ideal failing number).

Table S2: **Empirical estimation for κ and γ for the 12 data categories.**

(a) Compressed audio

κ \ γ	1/2	1/4	1/8	1/16	1/32
1/2	0.1408	0.1330	0.1596	0.2660	0
1/4	0.1596	0.1596	0.1330	0.5319	0
1/8	0.1064	0.1330	0	0	0
1/16	0.2660	0.5319	0	0	0
1/32	0	0	0	0	0

* Table entries are percentages; e.g. the first entry means $0.1408\% = 0.001408$.

(b) Audio

κ \ γ	1/2	1/4	1/8	1/16	1/32
1/2	0.1698	0.1164	0.1400	0.1182	0.1773
1/4	0.0887	0.0560	0.0532	0.1330	0.5319
1/8	0.0332	0	0	0	0
1/16	0	0	0	0	0
1/32	0	0	0	0	0

(c) Compressed video

κ \ γ	1/2	1/4	1/8	1/16	1/32
1/2	0.1964	0.1272	0.1533	0.1330	0.1330
1/4	0.1850	0.1544	0.2013	0.1478	0.0887
1/8	0.1983	0.1869	0.2660	0.1773	0
1/16	0.2161	0.1182	0.1773	0.2660	0
1/32	0.2660	0.2660	0.3546	1.0638	0

(d) Video

κ \ γ	1/2	1/4	1/8	1/16	1/32
1/2	0.2579	0.2716	0.2482	0.2327	0.2660
1/4	0.2490	0.2992	0.3639	0.4137	0.5319
1/8	0.2660	0.3080	0.4255	0.5319	1.0638
1/16	0.1995	0.1773	0.1330	0	0
1/32	0.3546	0.3546	0	0	0

(e) Compressed image

κ \ γ	1/2	1/4	1/8	1/16	1/32
1/2	0.2872	0.3224	0.3919	0.2364	0.1773
1/4	0.2095	0.2128	0.2660	0.2660	0
1/8	0.2520	0.2128	0.3989	1.0638	0
1/16	0.1182	0	0	0	0
1/32	0	0	0	0	0

(f) Image

κ \ γ	1/2	1/4	1/8	1/16	1/32
1/4	0.4110	0.4074	0.3191	0.3324	0.4433
1/8	0.3622	0.3823	0.2520	0.3546	0.1773
1/16	0.3723	0.4199	0.3191	0.6649	0.5319
1/32	0.3657	0.4728	0.1330	0	0
1/64	0.4433	0.7092	0	0	0

(g) Compressed social network data

κ \ γ	1/2	1/4	1/8	1/16	1/32
1/2	0.2176	0.2037	0.2482	0.3657	0.3546
1/4	0.1924	0.1995	0.2520	0.4728	0.7092
1/8	0.2305	0.2240	0.2660	0.5319	0.5319
1/16	0.2327	0.2364	0.2660	0.5319	
1/32	0.0887	0	0	0	0

(h) Social network data

κ \ γ	1/2	1/4	1/8	1/16	1/32
1/8	0.3191	0.3137	0.3324	0.3989	0.2660
1/16	0.3103	0.3103	0.3682	0.4255	0
1/32	0.2128	0.3103	0.3191	0.5319	0
1/64	0.1330	0.1330	0	0	0
1/128	0	0	0	0	0

(i) Compressed DNA sequenced data

κ \ γ	1/2	1/4	1/8	1/16	1/32
1/2	0.1854	0.2150	0.1596	0.1995	0.2660
1/4	0.1471	0.1662	0.1120	0.1182	0
1/8	0.1418	0.1680	0.1064	0.1330	0
1/16	0.1662	0.2364	0	0	0
1/32	0.2660	0.3546	0	0	0

(j) DNA sequenced data

κ \ γ	1/2	1/4	1/8	1/16	1/32
1/8	0.2579	0.2660	0.1596	0	0
1/16	0.2240	0.1596	0	0	0
1/32	0.2364	0.2660	0	0	0
1/64	0	0	0	0	0
1/128	0	0	0	0	0

(k) Compressed text

κ \ γ	1/2	1/4	1/8	1/16	1/32
1/2	0.3707	0.3961	0.3191	0.2327	0.3546
1/4	0.2943	0.3158	0.3080	0.3546	0.7092
1/8	0.1950	0.2520	0.2128	0.2660	0
1/16	0.1662	0.2364	0.1330	0	0
1/32	0	0	0	0	0

(l) Text

κ \ γ	1/2	1/4	1/8	1/16	1/32
1/8	0.2812	0.2561	0.2240	0.2313	0.1064
1/16	0.2766	0.2660	0.2455	0.2837	0.0887
1/32	0.2402	0.2081	0.2128	0.2660	0.1773
1/64	0.1900	0.2128	0.1773	0.3546	0
1/128	0	0	0	0	0

For every data category we realize the empirical estimation method (p. 4). Recall that the selected acceptable (κ, γ) pair is the top-left corner of the consistent subtable where the percentage of failures is smaller than 0.25% (slightly below the ideal 0.27% for NIST). Samples used for parameter estimation are not used in any other part of the experiment.

Table S3: **Extraction quality evaluation by NIST.**

Data category (compressed)	Audio		Video		Images		Social network		DNA sequenced		Text	
Sample size	1.5 GB		7.3 GB		4.8 GB		5 GB $\times 4$		2.9 GB		12 GB $\times 4$	
NIST TEST SUITE	**Prop.**	**$\mathcal{P}$-val**	**Prop.**	**$\mathcal{P}$-val**	**Prop.**	**$\mathcal{P}$-val**	**Prop.**	**$\mathcal{P}$-val**	**Prop.**	**$\mathcal{P}$-val**	**Prop.**	**$\mathcal{P}$-val**
Frequency	0.9914	0.090936	0.9853	0.474986	0.9960	0.334538	0.9923	0.048716	0.9880	0.047173	0.9927	0.022760
Block frequency	0.9914	0.075719	0.9853	0.213309	0.9933	0.001824	0.9910	0.129620	0.9907	0.048716	0.9863	0.042808
Cumulative sums	0.9957	0.213309	0.9853	0.401199	0.9960	0.155936	0.9913	0.070081	0.9913	0.307818	0.9928	0.020548
Runs	0.9943	0.719747	0.9907	0.275709	0.9920	0.075719	0.9903	0.021262	0.9947	0.062821	0.9900	0.062821
Longest run	0.9943	0.080519	0.9853	0.045675	0.9947	0.003851	0.9873	0.026948	0.9867	0.047173	0.9897	0.230755
Rank	0.9800	0.001399	0.9907	0.249284	0.9933	0.068999	0.9893	0.030806	0.9893	0.419021	0.9923	0.001296
FFT	0.9886	0.057146	0.9813	0.621506	0.9893	0.042808	0.9883	0.055361	0.9933	0.145326	0.9913	0.057146
Non-overlapping template	0.9898	0.000101	0.9899	0.011585	0.9900	0.003084	0.9897	0.000274	0.9901	0.001895	0.9903	0.000544
Overlapping template	0.9971	0.262249	0.9920	0.574903	0.9933	0.155936	0.9890	0.090936	0.9920	0.268917	0.9837	0.026948
Universal	0.9800	0.605916	0.9800	0.509162	0.9827	0.068999	0.9853	0.035174	0.9880	0.213309	0.9887	0.015598
Approximate entropy	0.9886	0.626709	0.9880	0.334538	0.9853	0.035174	0.9903	0.032923	0.9920	0.595549	0.9887	0.078086
Random excursions	0.9863	0.000533	0.9866	0.048716	0.9899	0.062821	0.9881	0.008016	0.9882	0.001949	0.9903	0.006483
Random excursions variant	0.9905	0.011250	0.9942	0.020548	0.9898	0.094664	0.9916	0.001738	0.9905	0.003201	0.9876	0.001001
Serial	0.9857	0.350485	0.9913	0.319084	0.9933	0.037566	0.9890	0.021999	0.9880	0.034031	0.9905	0.062821
Linear complexity	0.9857	0.021262	0.9947	0.202268	0.9867	0.549331	0.9893	0.181557	0.9853	0.008491	0.9913	0.129620

Data category (uncompressed)	Audio		Video		Images		Social network		DNA sequenced		Text	
Sample size	9GB		3.7 GB		3.8 GB		5 GB $\times 3$		8 GB		20 GB $\times 2$	
NIST TEST SUITE	**Prop.**	**$\mathcal{P}$-val**	**Prop.**	**$\mathcal{P}$-val**	**Prop.**	**$\mathcal{P}$-val**	**Prop.**	**$\mathcal{P}$-val**	**Prop.**	**$\mathcal{P}$-val**	**Prop.**	**$\mathcal{P}$-val**
Frequency	0.9960	0.060875	1.0000	0.073417	0.9907	0.022760	0.9911	0.105618	0.9880	0.554420	0.9927	0.035174
Block frequency	0.9920	0.699313	0.9867	0.595549	0.9820	0.514124	0.9942	0.030297	0.9880	0.045675	0.9940	0.027868
Cumulative sums	0.9940	0.023545	0.9987	0.204985	0.9953	0.055361	0.9898	0.096578	0.9853	0.358641	0.9917	0.099513
Runs	0.9907	0.474986	0.9960	0.437374	0.9973	0.081760	0.9884	0.093720	0.9907	0.068999	0.9920	0.268917
Longest run	0.9947	0.075719	0.9947	0.108791	0.9920	0.262249	0.9871	0.034031	0.9893	0.002512	0.9873	0.176657
Rank	0.9867	0.678666	0.9907	0.026948	0.9880	0.092319	0.9898	0.016149	0.9933	0.073417	0.9827	0.089572
FFT	0.9840	0.153763	0.9867	0.383827	0.9880	0.304126	0.9893	0.003712	0.9800	0.042118	0.9847	0.149495
Non-overlapping template	0.9897	0.000195	0.9899	0.003996	0.9898	0.004462	0.9900	0.000070	0.9901	0.002559	0.9904	0.000057
Overlapping template	0.9840	0.000216	0.9813	0.569766	0.9920	0.272297	0.9867	0.122325	0.9827	0.153763	0.9827	0.045675
Universal	0.9907	0.423545	0.9971	0.042808	0.9960	0.779188	0.9867	0.075719	0.9853	0.135331	0.9880	0.118812
Approximate entropy	0.9907	0.194289	0.9933	0.075719	0.9880	0.066882	0.9902	0.102526	0.9933	0.451234	0.9947	0.042808
Random excursions	0.9872	0.005930	0.9863	0.030806	0.9931	0.103401	0.9867	0.003577	0.9883	0.025522	0.9913	0.001691
Random excursions variant	0.9885	0.010817	0.9881	0.017912	0.9920	0.007616	0.9900	0.000895	0.9901	0.009509	0.9919	0.004681
Serial	0.9900	0.282626	0.9880	0.124115	0.9873	0.275709	0.9920	0.081760	0.9867	0.414525	0.9900	0.010606
Linear complexity	0.9920	0.162606	0.9813	0.275709	0.9880	0.075719	0.9920	0.191687	0.9947	0.059923	0.9933	0.213309

The input samples to RRB are files of size 1.5 GB – 20 GB from 12 data categories, whenever there is no large enough file we concatenate (potentially correlated) smaller files together. Sample sizes are listed for every data category and RRB is applied independently on enput sample with parameters estimated in Table S2. For every run of RRB the extraction quality is evaluated by the NIST test suite for 4 times with the test input equally partitioned into 50, 100, 200, and 400 subsequences respectively. NIST tests compute $\mathcal{P}$-values for every subsequence in every test and then apply a χ^2 test to calculate a second-order $\mathcal{P}$-value. The overall proportion of subsequences with $\mathcal{P}$-value ≥ 0.01 and the *minimum* second-order $\mathcal{P}$-values are reported. To pass one NIST test, the second-order $\mathcal{P}$-value should be greater than 0.0001, and the proportion of subsequences with $\mathcal{P}$-value ≥ 0.01 must be greater than 0.940, 0.960, 0.965, and 0.975 respectively for 50 – 400 subsequences. The second-minimum $\mathcal{P}$-value of "non-overlapping template" tests is 0.001895 for uncompressed social data and 0.003511 for uncompressed text. The reported $\mathcal{P}$-value is *not* uniformly distributed, since it is the minimum among multiple NIST tests.

Table S4: **Extraction quality evaluation by DIEHARD.**

Data category (compressed)	Audio	Video	Images	Social network	DNA sequenced	Text
DIEHARD SUITE	**$\mathcal{P}$-val**	**$\mathcal{P}$-val**	**$\mathcal{P}$-val**	**$\mathcal{P}$-val**	**$\mathcal{P}$-val**	**$\mathcal{P}$-val**
Birthday spacings	0.438088	0.310881	0.058193	0.554324	0.808198	0.000052
Overlapping permutations	0.752959	0.428180	0.639592	0.723372	0.056572	0.243835
Ranks of 31x31 matrices	0.569184	0.339979	0.920656	0.482406	0.385041	0.362155
Ranks of 32x32 matrices	0.547909	0.403446	0.613285	0.433728	0.352847	0.321909
Ranks of 6x8 matrices	0.645499	0.845557	0.345142	0.018670	0.701125	0.881740
Bit stream test	0.387205	0.593575	0.014402	0.654673	0.125810	0.431669
Monkey tests OPSO	0.355216	0.197500	0.667192	0.914133	0.238029	0.572953
Monkey tests OQSO	0.645485	0.669454	0.480169	0.629274	0.027371	0.834129
Monkey tests DNA	0.486269	0.940092	0.174359	0.591367	0.874612	0.858750
Count 1's in a stream of bytes	0.608089	0.917741	0.427466	0.822289	0.726707	0.597594
Count 1's in specific bytes	0.714184	0.097330	0.151793	0.462850	0.294159	0.656062
Parking lot test	0.152996	0.151125	0.687893	0.119339	0.251395	0.044892
Minimum distance test	0.897483	0.896119	0.638546	0.846002	0.654949	0.466783
Random spheres test	0.546443	0.057325	0.295651	0.501201	0.782601	0.678879
The squeeze test	0.541244	0.436086	0.144311	0.721372	0.942856	0.919760
Overlapping sums test	0.240819	0.239792	0.053650	0.577147	0.055292	0.307435
Runs test (up)	0.962195	0.537571	0.269606	0.671718	0.411167	0.079167
Runs test (down)	0.813418	0.358613	0.321261	0.837789	0.472629	0.807418
Craps test no. of wins	0.642862	0.586490	0.113479	0.737114	0.252371	0.015010
Craps test throws per game	0.276496	0.697953	0.028449	0.927459	0.278761	0.611609

Data category (uncompressed)	Audio	Video	Images	Social network	DNA sequenced	Text
DIEHARD SUITE	**$\mathcal{P}$-val**	**$\mathcal{P}$-val**	**$\mathcal{P}$-val**	**$\mathcal{P}$-val**	**$\mathcal{P}$-val**	**$\mathcal{P}$-val**
Birthday spacings	0.385522	0.828963	0.004437	0.208048	0.057008	0.955554
Overlapping permutations	0.724376	0.512534	0.985741	0.209806	0.863579	0.918563
Ranks of 31x31 matrices	0.842685	0.636229	0.357475	0.881277	0.600983	0.409768
Ranks of 32x32 matrices	0.822334	0.503910	0.890231	0.619142	0.419850	0.576953
Ranks of 6x8 matrices	0.591588	0.563394	0.957945	0.013593	0.175439	0.339026
Bit stream test	0.155471	0.321259	0.228977	0.527317	0.863584	0.206301
Monkey tests OPSO	0.630120	0.714526	0.012538	0.058461	0.534801	0.723825
Monkey tests OQSO	0.398779	0.380577	0.955060	0.251407	0.694832	0.169836
Monkey tests DNA	0.862675	0.260406	0.576408	0.890297	0.507449	0.823096
Count 1's in a stream of bytes	0.452006	0.915969	0.211816	0.616283	0.206299	0.192805
Count 1's in specific bytes	0.283858	0.819726	0.257865	0.244149	0.763107	0.782839
Parking lot test	0.020669	0.256870	0.590298	0.293398	0.931840	0.076152
Minimum distance test	0.467898	0.851627	0.267924	0.343415	0.767186	0.734031
Random spheres test	0.316626	0.077993	0.473525	0.700093	0.822260	0.033127
The squeeze test	0.922929	0.258466	0.260324	0.584267	0.434682	0.751415
Overlapping sums test	0.001460	0.030426	0.074745	0.009624	0.034810	0.080606
Runs test (up)	0.185805	0.954631	0.998131	0.516886	0.526526	0.637137
Runs test (down)	0.034008	0.365320	0.967070	0.368460	0.390886	0.759504
Craps test no. of wins	0.032640	0.979268	0.068112	0.537164	0.856300	0.259566
Craps test throws per game	0.018952	0.675906	0.882955	0.428987	0.757852	0.280388

The test inputs for DIEHARD are the same extracted outputs as in Table S3. DIEHARD computes $\mathcal{P}$-values for every extracted output in every statistical test. When there are multiple $\mathcal{P}$-values in one test DIEHARD uses a Kolmogorov-Smirnov (KS) test to obtain a final $\mathcal{P}$-value. In case there are multiple final $\mathcal{P}$-values the first obtained one is reported. The test passes if the final $\mathcal{P}$-value is in $[0.01, 0.99]$.

Table S5: **Data used in experiments.**

Description	Url	Remark
**Social network** (compressed and uncompressed)		
Stackoverflow data dump	`https://archive.org/download/stackexchange`	Last download: 2014-09-26 / plain text
Amazon product co-purchasing network metadata	`https://snap.stanford.edu/data/amazon-meta.html`	Product meta data and reviews / plain text
**Text** (compressed and uncompressed)		
Linguistic Data Consortium (LDC). Data sources: varied, web collection,telephone speech, newswire news magazine, journal articles, continuous speech corpus	`https://catalog.ldc.upenn.edu`	Plain text (untagged) and text/words tagged with statistics DVDs: 1–6
**Images** (compressed and uncompressed)		
Astronomy picture of the day	`http://apod.nasa.gov/apod/astropix.html`	Last download: 2014-9-13 / (original) jpg From: abell1185_cfht_big.jpg To: zvezdalaunch_nasa.jpg
**Audio** (compressed and uncompressed)		
Project Gutenberg	`http://www.gutenberg.org/browse/categories/1`	Last download: 2014-12-14 / (original) m4b All
**DNA sequences** (compressed and uncompressed)		
Assembled human genomes from NCBI	`ftp://ftp.ncbi.nih.gov/genomes/H_sapiens /Assembled_chromosomes/`	From: hs_alt_CHM1_1.1_chr10.fa.gz To: hs_alt_HuRef_unplaced.mfa.gz

**Video** (compressed and uncompressed) From: `https://www.youtube.com`		
Video title	Video title	Video title
A Bridge Too Far 1977 - Full Movie	Alfred Hitchcock - Notorious	Don't Come Knocking 2005 - Wim Wenders
Fear and Desire (Restored Full Length) (1953) - Kubrick	Hercules	Star Wars Threads of Destiny (2014)
The Complete Citizen Kane - Documentary	The Phantom Edit	Waterloo (1970)
Zulu (1964) PL	David And Goliath - Orson Welles	Inside the Making of Dr. Strangelove
Jerry Seinfeld - I'm telling you for the last time	laberinto de pasiones 1982 - Pedro Almodovar	Live concert Rachmaninov - Vespers
Manu Chao - Clandestino Full Album HD	Manos Hatzidakis (A walk on the moon)	Pirates of the Caribbean Soundtrack
Ramones - Live In Cologne, Gemany 92 full concert	Rimsky-Korsakov Sheherazade - J.Suk, Czech PO, A.Rahbari	Seinfeld How It Began (full)
Star Trek Friendship One	Star Trek full episode season 3 episode 20	Star Trek The Enterprise Incident
Star Trek Mirror season 2 episode 10	Star Trek the devil in the dark season 1 episode 26	Star Trek Voyager - The Borg Arc
The Best of Debussy	The Sex Pistols Live At Winterland San Fransisco 1978	Until The End Of The World - Wim Wenders
Woody Allen - Wild Man Blues (1997)	Roman Market (Manos Hatzidakis)	Amélie - Full Soundrack
A mi madre le gustan las mujeres	Charlie Chaplin The Kid - Film 1921	Citizen Kane Anatomy Of A Classic

2 Mathematical analysis

2.1 Summary of theoretical results

We provide general versions of our definitions and theorems including full proofs. For definitions and notation see Section 2.3. The full parameters of our results are listed in Table S6.

Construction. We present a randomness extractor, which is a streaming algorithm that uses local memory of size $\mathsf{polylog}(n)$, a $\mathsf{polylog}(n)$ random seed, and two external read/write streams (tapes). This extractor makes $\mathrm{O}\left(\log(\log \frac{n}{\varepsilon})\right)$ many passes to produce an output of statistical distance at most ε to the uniform distribution for input sources of min-entropy $\Omega(n)$. We obtain the following provable guarantees.

i. *Arbitrary sources.* Our streaming algorithm provably extracts $m = \Omega\left(n\right)$ many bits from one length n sample.

ii. *Affine and bit-fixing sources.* The same streaming algorithm works for affine and bit-fixing sources with better parameters.

For the typical case where $\varepsilon = 1/\mathsf{poly}(n)$ the local memory size and seed length both are $\mathrm{O}\left(\log^2 n\right)$, and the number of passes is $\mathrm{O}\left(\log\log n\right)$.

Lower bounds. We prove that this log-log in the number of passes is tight.

iii. We show that every multi-stream extractor that uses local memory of size $s = n^{o(1)}$ must make $\Omega\left(\log\log\frac{1}{\varepsilon}\right)$ many passes. This holds even when given any constant number of independent bit-fixing sources, which is a stronger statement. The proof introduces mathematical machinery, which may be interesting in its own right.

iv. We say that an extractor is *oblivious* if, after fixing the bits of the seed, the head-move on the streams depends only on the input length. We show that for sources of min-entropy at most $n^{1-\delta}$ where $\delta > 0$ is constant, every oblivious extractor with memory $n^{o(1)}$ must make $\Omega\left(\log n\right)$ many passes. This is exponentially bigger than the number of passes in our construction. Note that every known general extractor (streaming or not) is oblivious.

Remark: We include the mathematics analysis and techniques for streaming randomness extraction for completeness. The theoretical analysis of the construction and the lower bounds involve a *number of new* mathematical techniques – also answering an open question about single-sample next-block min-entropy. This is a very involved part of our work, it can be read independently of the extensive empirical methods and study (see main body) and is listed here only for completeness.

2.2 Theoretical background and our techniques

Randomness extraction. For a concise overview of the mathematics of randomness extraction see [21] and references within. The most common type of extractor gets two inputs: one from an arbitrary statistical source $X \subseteq \{0,1\}^n$ (aka weak source) and the other is a uniformly distributed random seed of length d (typically $d = \mathsf{polylog}(n)$). The extractor outputs an m-bit long string, which has statistical distance to the uniform distribution at most ε. The only and minimal requirement is that X has min-entropy at least k. Other than this the same extractor should work for every source, including those overtaken by adversaries. This type of *seeded extractor*, or *extractor*, was introduced in [34]. There are two more important types of extractors, the *multiple-independent-source* extractor [39, 27, 40, 41, 42] and the *deterministic* (or seedless) extractor. Although, deterministic extraction is not in general feasible, it becomes possible for restricted families of sources with prominent examples that of *bit-fixing* (some bits are fixed and the rest uniformly iid) and *affine sources* (all the support in an affine subspace) [43, 44, 45, 46]. The

bulk of previous work considered polynomial time efficient extractors and studied in detail the relation between the parameters mentioned above: n, m, d, k, and ε. In this work we require stronger efficiency, where "efficient" means "computable by a streaming algorithm", i.e. a big source extractor. Whenever we talk about "big sources" the term "big" quantifies the sample size relative to the employed computational resources. The daunting question is whether streaming extraction is at all feasible.

Example of a streaming-friendly setting. Two early works in randomness extraction [27, 39] dealt with the following restricted family of sources, known as Santha-Vazirani (SV) sources. A source $X \in \{0,1\}^n$ has guaranteed next-block-min-entropy α, if X is partitioned into b blocks $X = X_1, \ldots, X_b$ and $\mathsf{H}_\infty[X_i \big| X_{i-1}, \ldots, X_1] \geq \alpha$ for all $i = 1, \ldots, b$. For simplicity, let n be a multiple of b and $|X_i| = n/b$. Here $\mathsf{H}_\infty[X_i \big| X_{i-1}, \ldots, X_1] = \min_{x_1,\ldots,x_i} \left\{\log\left(1/\Pr[X_i = x_i \big| X_{i-1} = x_{i-1}, \ldots, X_1 = x_1]\right)\right\}$ is the *next-block-min-entropy of block i*. That is, block i has enough min-entropy left even conditioned on a worst-case choice of values of blocks $1, \ldots, i-1$. Such sources have been scrutinized for years; see e.g. [26, 27, 39] and its recent pseudorandom analogs [47, 48]. Since the sources have next-block-min-entropy, almost uniform random bits can be extracted by applying the *same extractor with the same seed* locally on every block. This extractor can be a 2-universal hash function h sampled using $\mathrm{O}(n/b)$ bits, and then a single-pass streaming algorithm computes $h(X_1), \ldots, h(X_b)$ by processing every block locally within $\mathrm{O}(n/b)$ memory. This is a single-stream and single-pass algorithm that extracts $\Omega(\alpha \cdot b)$ bits.

This theoretical algorithm relies on independence assumptions about the weak source and hence it cannot apply to general weak sources [9]; see also, Table **??** for a real-world experimental study. One way to use this extractor in practice is by concatenating b many independent samples. However, b has to be polynomial in n since the local memory is $\mathsf{polylog}(n)$.

Streaming settings and parameters. On the positive side our main contribution is a two-stream seeded extractor, which we call the *Random Re-Bucketting* (RRB) algorithm. This is the first construction shown to extract randomness in important settings, while at the same time is perhaps the simplest extractor conceptually that appeared in the literature.

The multi-stream setting commonly models a hard disk or disk array as a stream [6, 7, 8]. Our paper initiates the theoretical study of streaming extraction in practically demanding settings. We observe that the standard streaming parameters (i.e. number of streams, local memory size, and number of passes) are insufficient to adequately formulate a streaming setting. For instance, although every logarithmic space computable function can be computed using two streams and $\mathrm{O}(\log n)$ many passes, the known construction [50, 51] is very non-practical. This is not only because we cannot afford $\mathrm{O}(\log n)$ passes, but mainly because the stream size blows up by a polynomial in n and thus the correspondence between streams and hard disks (or any other external storage) is lost. Besides the stream size, we also consider the issue of head-move policy (see also the random cyclic shift RCS extractor in the appendix), and study the issue of oblivious (the head position is only a function of the time step) vs non-oblivious head-move. Table S6 summarizes our results for these parameters.

For a comprehensive treatment of parameters, we note that one can beat the $\Omega\left(\log\log\frac{1}{\varepsilon}\right)$ bound in the number of passes by using a very long and thus impractical seed. In particular, using techniques from [6] we can construct (see Appendix 2.6.3 for details) a two-stream extractor that makes a constant number of passes and uses a seed of length $d = n^2$ to extract $m = \Omega(k) + n^2$ many bits.

Our techniques and comparison to previous work. For single-stream algorithms, thirteen years ago Bar-Yossef et al. [9] (see also [52]) showed that it is impossible to extract randomness from bit-fixing sources by making a single pass over the stream. The same lower bound generalizes to algorithms that use a single read/write stream over which they make $\Omega(n/s)$ many passes using local memory of size s.

Proving lower bounds for the multi-stream model is a much more involved task since known crossing sequence-like or communication complexity arguments break down. Note that no $\omega(\log n)$ lower bound can be obtained since

Extractors				
**algorithm**	**# passes**	**seed length d**	**output length m**	**max stream size**
RRB	$\mathrm{O}\left(\log\log\frac{n}{\varepsilon}\right)$	$\mathrm{O}\left(\log n\cdot\log\frac{n}{\varepsilon}\right)$	$\Omega\left(n\right)$	$\mathrm{O}\left(n\right)$
* RCS (on bit-fixing source)	$\mathrm{O}\left(\log\log\frac{n}{\varepsilon}\right)$	$\mathrm{O}\left(\log^2 n\cdot\log\frac{1}{\varepsilon}\right)$	$\Omega\left(n\right)$	$\mathrm{O}(n\log\frac{1}{\varepsilon})$

Lower Bounds				
**setting**	**# passes**	**seed length d**	**output length m**	**head move policy**
general extractor	$\Omega\left(\log\log\frac{1}{\varepsilon}\right)$	$\mathrm{O}\left(s\right)$	$s\cdot\mathsf{polylog}(\frac{1}{\varepsilon})$	arbitrary
oblivious extractor	$\Omega\left(\log n\right)$	$\leq m^{0.99}$	$s\cdot\mathsf{poly}(n)$	oblivious for any fixed seed

Table S6: The local memory size is denoted by s, which is $\mathsf{polylog}(n)$ in RRB and RCS. The weak source X has min-entropy $k=\Omega\left(n\right)$, except from the oblivious lower bound where $k=n^{1-\Omega(1)}$, e.g. $k=n^{0.99}$. * RCS has constant stream speed, i.e. the heads on all streams progress after the same fixed number of time steps.

[50] and [53] imply an $\mathrm{O}\left(\log n\right)$-pass extractor (but with an unrealistic stream size). We develop a technique to study statistical dependencies in a multi-stream setting. This differs from lower bounds proved for big pseudorandom objects [6] – an extractor has an output smaller than its input, whereas for pseudorandom objects the lower bound crucially relies on the fact that the input is stretched in the output. Our lower bounds are inspired by the framework of [7, 8, 49] with the important difference that these previous works aim to prove streaming lower bounds for single problems, whereas a lower bound for an extractor must hold *simultaneously* for *all* output distributions that are ε-close to uniform. Note that our lower bound holds even for bit-fixing sources, and in fact for any constant number of bit-fixing sources; i.e. this lower bound applies to extractors that are simultaneously seeded and multiple-independent-source.

A technical problem in realizing a streaming extractor is that the multi-stream model is inherently limited in permuting input blocks. This is true even when the head can move in two directions over the streams [7, 8, 49]. Our slightly-above-constant lower bound implies that at least a mild form of permutation is necessary. Moreover, by putting in perspective all previous constructions (e.g. [54, 55, 22]) we observe that they all access the input bits in a permuted fashion, and this is true even after fixing the seed. These previous extractors make expensive operations when viewed as streaming algorithms. The same holds true for multi-source extractors, where access to the input also relies on accessing the input through streaming-wise-costly combinatorial objects such as error-correcting codes [41, 42]; e.g. multiplying a matrix M by a vector x can be easily shown [8] to require $\Omega\left(\log n\right)$ many passes or polynomial local memory.

Conceptually, our extractor differs from known seeded extractors, e.g. [22] and its followups. The first stages of RRB transform an arbitrary weak source into a source with guaranteed next-block-min-entropy. Based on an involved argument (where much of our technical effort was spent), such transformation can be realized with "streaming friendly" permutations, which cost only $\mathrm{O}\left(\log\log\frac{n}{\varepsilon}\right)$ passes when implemented appropriately. The last stage of RRB is an one-pass local extractor that works correctly for guaranteed next-block-min-entropy (also used in [26, 27, 39]). Finally, we note that RRB is an oblivious streaming extractor (i.e. after fixing the seed the head-move on the external streams depends only on n). Thus, RRB, which makes $\mathrm{O}\left(\log\log\frac{n}{\varepsilon}\right)$ passes, does not work for sources of min-entropy $k=o\left(n\right)$, where $\Omega\left(\log n\right)$ many passes are needed.

2.3 Notation and terminology

We introduce notation common throughout the paper.

Probability notation. We use capital letters to denote random variables and sets, and in particular X,Y,Z for random variables and S,T for sets. For convenience we let $[n]=\{1,2,3,\ldots,n\}$. We denote types of independent

random variables by calligraphic letters such as $\mathcal{U}_n$ for the uniform distribution over n elements. All distributions are over $\{0,1\}^n$ for some positive integer n. The statistical distance between two distributions X, Y over $\{0,1\}^n$ is defined as $\mathcal{SD}(X,Y) = \frac{1}{2}\sum_{w\in\{0,1\}^n} \left|\Pr[X=w] - \Pr[Y=w]\right|$. We say that X is *ε-close to uniform* if $\mathcal{SD}\left(X, \mathcal{U}_{|X|}\right) \le \varepsilon$.

Lowercase letters such as x, y are used for binary strings. By subscripting a string with a set, e.g. $x|_S$, we denote the subsequence of x restricted on the positions specified by S in increasing order, and the same holds for random variables. For example, $x|_{\{1,3,42\}} = (x_1, x_3, x_{42})$. Substrings of x are denoted by $\overline{x}_1, \overline{x}_2, \cdots$, and in particular $(\overline{x}_1, \ldots, \overline{x}_b)$ refers to a partition of x into b many consecutive parts, where each part $\overline{x}_i$ is called a *block of x*. Moreover, when x is sampled from a random variable X, for every $i \in [b]$ we denote by $\overline{X}_i$ the block of X where $\overline{x}_i$ is sampled from. To avoid ambiguity, brackets are used to index bits; e.g. $X_1[2]$ denotes the second bit of the random variable X_1.

If a random variable X takes values over S, then its *entropy* (or Shannon entropy) is defined as $\mathsf{H}[X] = -\sum_{x\in S}\Pr[X=x]\log(\Pr[X=x])$, and its *min-entropy* is defined as $\mathsf{H}_\infty[X] = -\log(\max_{x\in S}\Pr[X=x])$, where all logarithms are of base 2. Two crucial quantities appearing in this work is that of (i) *conditional entropy*, where for Y taking values over S is defined as $\mathsf{H}[X|Y] = \sum_{y\in S}\Pr[Y=y]\mathsf{H}[X|Y=y]$, and (ii) *conditional min-entropy*, defined as $\mathsf{H}_\infty[X|Y] = \min_{y\in S}\mathsf{H}_\infty[X|Y=y]$. Note that conditional entropy is an averaging conditional quantity, whereas conditional min-entropy is a worst-case conditional statistical quantity.

An *(n,k)-source* X is a distribution over $\{0,1\}^n$ such that every random variable X satisfies $\mathsf{H}_\infty[X] \ge k$. X is an *(n,k)-bit-fixing source*, if there exists $y \in \{0,1\}^{n-k}$ and $T \subseteq [n], |T| = k$ such that X_T is distributed according to $\mathcal{U}_k$ and $X_{[n]\setminus T} = y$ is constant. We say that X is an *(n,k)-affine source* if X is uniformly distributed over a k-dimensional affine subspace of $\{0,1\}^n$. That is, $X = X' \cdot \mathbf{A} + \mathbf{b}$ for a fixed matrix $\mathbf{A} \in \{0,1\}^{k\times n}$ and row vector $\mathbf{b} \in \{0,1\}^n$, and a k-dimensional random row vector $X' \sim \mathcal{U}_k$.

2-Universal hash functions. $H = \{h : \{0,1\}^n \to \{0,1\}^m\}$ is *a family of 2-universal hash functions* if:

(i) for every $h \in H$, h is efficiently computable and its description is $\mathsf{poly}(n,m)$, where $\mathsf{poly}(n,m)$ denotes a function polynomial in n and m ;

(ii) for every $\mathbf{x}, \mathbf{y} \in \{0,1\}^n$ and $\mathbf{x} \ne \mathbf{y}$, $\Pr_{h\in H}[h(\mathbf{x}) = h(\mathbf{y})] = 2^{-m}$, where h is sampled uniformly at random from H.

Streaming algorithms. An (s,t,p) *streaming algorithm* is a Turing Machine that has local (internal) memory of size $s = s(n)$, and $t = t(n)$ unbounded external read/write streams, which are Turing Machine tapes that can be scanned from left to right for at most $p = p(n)$ many passes in total. Typically, we consider $s = \mathsf{polylog}(n)$, $t = \mathrm{O}(1)$, and $p = o(\log n)$. The requirement for sub-logarithmic number of passes makes the problem practical but also non-trivial since $(s,t,p) = (\mathrm{O}(\log n), 2, \mathrm{O}(\log n))$ is sufficient for every log-space[1] computable function[50, 51]. A function is *streaming computable* if it can be evaluated by a streaming algorithm. Whenever (s,t,p) is omitted then the previous typical values are assumed.

Randomness extractors. A *randomness extractor* $\mathsf{Ext} : \{0,1\}^n \times \{0,1\}^d \to \{0,1\}^m$ takes two inputs. The first input is a sample from the source and the second input is the seed. We say that Ext *is a (k,ε)-extractor* if for every (n,k)-source X and a uniformly random seed its output is ε-close to uniform, i.e. $\mathcal{SD}(\mathsf{Ext}(X,Y), Z) \le \varepsilon$, where $Y \sim \mathcal{U}_d$ and $Z \sim \mathcal{U}_m$. Ext is a (k,ε)-extractor for bit-fixing sources (or affine sources) if $\mathcal{SD}(\mathsf{Ext}(X,Y), Z) \le \varepsilon$ for every (n,k)-bit-fixing source (resp. (n,k)-affine source) X. Furthermore, Ext is a *streaming extractor* if it is streaming computable.

[1] Recall that a log-space computable function is one that can be computed by a Turing Machine of $\mathrm{O}(\log n)$ local memory size, one read-only input tape that can be read *polynomially* many times, and a write-only output tape [56].

A *streaming extractor* is a streaming algorithm where (i) the sample from the source is written on the first stream in the beginning of the computation, and (ii) the random seed of size $\mathsf{polylog}(n)$ is stored in the local memory. In all of our lower bounds we consider free access to the seed (regardless of size), which makes the lower bounds stronger.

2.4 The random re-bucketting (RRB) extractor

Here we present the RRB extractor together with its streaming implementation. This extractor makes $\mathrm{O}\left(\log\log\frac{n}{\varepsilon}\right)$ many passes. We also present its first provable property, which is that it extracts randomness from bit-fixing sources. RRB is a parametric algorithm. In the next section, we will see that with different parameters RRB enjoys next-block-entropy guarantees and hence works for general weak sources.

At a high-level, RRB consists of the following three stages.

I. Partition the n-bit long input into $b = \mathrm{O}\left(\log_2\frac{n}{\varepsilon}\right)$ many *super-blocks* each of length $\frac{n}{b}$. Inside each super-block choose uniformly and independently a random point to do the cyclic shift.

II. Re-bucket the b *super-blocks* into $\frac{n}{b}$ many *blocks* each of size b, where the i-th block consists of the i-th bit from every super-block, for $i = 1, 2, \ldots, \frac{n}{b}$.

III. Uniformly at random sample h from a 2-universal hash family. Function h can be a random Toeplitz matrix [57]. Then, apply the same h on the first $b_O = \Omega\left(\frac{n}{b}\right)$ many blocks, and output the result.

Note that only stage I and III use randomness, whereas stage II performs a fixed but "streaming-friendly" permutation. Therefore, the random seed y has the following form $y = \langle \underbrace{h}_{\text{hash function}}, \underbrace{r_1, \ldots, r_b}_{\text{indices for cyclic shifts}} \rangle$. The final local extraction can be performed in-place since the blocks are of size $b = \mathrm{O}\left(\log\frac{n}{\varepsilon}\right)$ which is sufficiently small.

Let us now introduce the shifting operator and after that we describe RRB.

Definition 1. *For $z = (z_1, \ldots, z_m) \in \{0,1\}^m$ and $r \in \{0, 1, 2, \ldots, m-1\}$, $\mathsf{shift}(z, r)$ denotes the string that obtained by cyclic shifting z to the left for r-bits, i.e. $\mathsf{shift}(z, r) = (z_{r+1}, z_{r+2}, \ldots, z_m, z_1, z_2, \ldots, z_r)$.*

For $n, k = \Omega(n)$ and $\varepsilon > 0$, the construction $\mathsf{RRB} : \{0,1\}^n \times \{0,1\}^d \to \{0,1\}^m$ has parameters $m = k - o(k)$, $b = \Theta(\log\frac{n}{\varepsilon})$ and $d = b + b\log\frac{n}{b}$, $b_O = \Omega\left(\frac{n}{b}\right)$. For convenience and without loss of generality, we further assume b to be a power of 2. Let σ_1, σ_2 denote the two streams and let $\sigma_2[j]$ denote the j-th bit in σ_2. Perhaps it helps to think of these parameters as follows.

Typical values

input length	n	output length	$m = \Omega(n)$
error tolerance	$\varepsilon = 1/n^{\Omega(1)}$	number of super-blocks	$b = \mathrm{O}(\log n)$
min-entropy	$k = \Omega(n)$	seed length	$d = \mathrm{O}\left(\log^2 n\right)$

Here is the description of RRB.

Algorithm: RRB

Data: $\sigma_1 \leftarrow x$, where x is an n-bit-long sample from weak source
Result: an m-bit-long binary string distributed ε-close to $\mathcal{U}_m$

Initialization:
$k \leftarrow$ lower bound on the min-entropy of the source
$y \leftarrow \mathcal{U}_d$ and partition $y = (h, y_1, \ldots, y_b) \in \{0,1\}^{2b} \times \left\{0, 1, 2, \ldots, \frac{n}{b} - 1\right\}^b$, where h specifies a 2-universal hash function $h : \{0,1\}^b \to \{0,1\}^{m/b_O}$ computable in $\mathrm{O}(\log n)$ space

Process:
1 partition $\sigma_1 = x = \left(\overline{x}_1, \overline{x}_2, \ldots, \overline{x}_b\right)$, where $|\overline{x}_i| = \frac{n}{b}$ for $1 \leq i \leq b$
2 $\sigma_2 \leftarrow (\mathsf{shift}(\overline{x}_1, y_1), \ldots, \mathsf{shift}(\overline{x}_b, y_b),)$
for $j = 1$ **to** $\left\lceil \log b \right\rceil$ **do**
3 $\quad \sigma_1 \leftarrow \left(\sigma_2[1], \sigma_2[\frac{n}{2}+1], \sigma_2[2], \sigma_2[\frac{n}{2}+2], \ldots, \sigma_2[\frac{n}{2}], \sigma_2[n]\right)$
4 $\quad \sigma_2 \leftarrow \sigma_1$
end
5 partition $\sigma_1 = \left(z_1, \ldots, z_{n/b}\right)$ where $|z_i| = b$ for $1 \leq i \leq \frac{n}{b}$
6 $\sigma_2 \leftarrow (h(z_1), \ldots, h(z_{b_O}))$

Output: σ_2

Claim 2. RRB *is streaming computable with* $\mathrm{O}(\log b)$ *passes over two streams and* $\mathrm{O}(b + \log n)$ *local memory.*

Proof. We analyze the number of passes and the usage of local memory stage-by-stage.

In Stage I (steps 1 and 2), step 2 makes four passes by copying the corresponding substrings (i.e. the super-blocks) from σ_1 to σ_2. In particular, in two passes write to σ_2 the left part of $\mathsf{shift}(\overline{x}_i, y_i)$ together with placeholders of the right part (i.e. $(\overline{x}_{i,y_i+1}, \overline{x}_{i,y_i+2}, \ldots, \overline{x}_{i,n/b}, *, ..., *)$) for $i \in [b]$; then invoke another two passes to fill in the placeholders with the rest of the y_i bits of $\mathsf{shift}(\overline{x}_i, y_i)$ (i.e. $\overline{x}_{i,1}, \ldots, \overline{x}_{i,y_i}$). Note, the latter does not need to store every y_i in memory, since y_i is already recorded implicitly as the length of the placeholders.

In Stage II (the for-loop), the iteration costs $\mathrm{O}(\log b)$ passes in total since the inner loop requires constant many passes: step 3 takes two passes over σ_1 and one pass over σ_2; step 4 is trivial and in fact it can be done by simply renaming σ_1 and σ_2 without any actual process over the streams.

Stage III (steps 5 and 6) is done using two passes in total. For every $i \in \left\{1, 2, \ldots, \frac{n}{b}\right\}$ it reads z_i from σ_1 into memory and then write $h(z_i)$ to σ_2. Since h is computable within $\mathrm{O}(\log n)$ space, $b + \mathrm{O}(\log n)$ local memory is sufficient to buffer z_i and compute $h(z_i)$.

Therefore, RRB uses $\mathrm{O}(\log b)$ passes over two streams σ_1, σ_2 and $\mathrm{O}(b + \log n)$ local memory. $\square$

2.4.1 Streaming extraction from bit-fixing sources

Theorem 3. *For every* $k = \Omega(n)$*, there exists* $b = \Theta(\log \frac{n}{\varepsilon})$*, such that for* $d = b + b \log \frac{n}{b}$ *and* $b_O = \frac{n}{b}$*,* RRB *is a* (k, ε)*-extractor for every* (n, k)*-bit-fixing source with* $m = k - o(k)$*.*

Proof. The proof relies on the following two lemmas. Lemma 4 asserts that the first two stages of RRB achieves next-block-min-entropy guarantee. Lemma 7 illustrates how the last stage of RRB extracts randomness from next-block-min-entropy.

Lemma 4. *Fix an arbitrary (n,k)-bit-fixing source X, $Y \sim \mathcal{U}_d$, and let random variable*[2] $Z = (Z_1, \ldots, Z_{n/b})$ *be the content of σ_1 in step 5 in the computation of $\mathsf{RRB}(X,Y)$. Then, for every $k = \Omega(n)$ and for every positive $\delta < \frac{k}{n}$, with probability greater than $1 - \frac{n}{b} \cdot \exp\left(-2\delta^2 b\right) = 1 - 2^{-\Omega(b)} n$ over the random choice of Y, all blocks in Z have next-block-min-entropy $\alpha = \left(\frac{k}{n} - \delta\right) b$.*

Proof of Lemma 4. We first analyze the structure of Z and then bound the probability that z_j has min-entropy $\Omega(kb/n)$. We conclude by union bound the next-block-min-entropy lower bound for the blocks in Z.

Since X is an (n,k)-bit-fixing source without loss of generality there exists $S \subseteq [n]$ consisting of k indices such that $X_S = \mathcal{U}_k$, whereas $X_{[n]\setminus S}$ is fixed. Let $S_j = S \bigcap \left\{\frac{n}{b}(j-1)+1, \frac{n}{b}(j-1)+2, \ldots, \frac{n}{b}\cdot j\right\}$ denote the indices of unfixed bits in the j-th input block for $j \in [b]$ (i.e. $\overline{x}_j$ in step 1 of RRB). Then, let $k_j = |S_j|$ and $k = \sum_{i=j}^{b} k_j$.

Now, we upper bound $\Pr\left[\mathsf{H}_\infty[Z_1] < \alpha\right]$, i.e. the probability over the random choice of Y that Z_1 does not have min-entropy $\alpha = \left(\frac{k}{n} - \delta\right) b$. Note that for every fixed Y, Z_1 is a deterministic projection of $X = (X_1, \ldots, X_n)$. Thus, its min-entropy equals to the number of unfixed bits from X. Therefore, it suffices to count the number of unfixed bits in Z_1.

For every $Y = y$ and y_j determined by y as in RRB, let $I_j = I_j(y_j)$ be the indicator that the j-th bit of Z_1 is not fixed. However, as long as b is a power of 2 the j-th bit of Z_1 is also the first bit in $\mathsf{shift}(\overline{X}_j, y_j)$, i.e. $Z_{1,j} = \mathsf{shift}(\overline{X}_j, y_j)_1 = (\overline{X}_j)_{y_j+1} = X_{\frac{n}{b}(j-1)+y_j+1}$. Thus, $\mathsf{H}_\infty[Z_1] = \sum_{j=1}^{b} I_j$ where for every $j \in [b]$, $I_j = 1$ if and only if $\frac{n}{b}(j-1) + y_j + 1 \in S$.

By definition, y_j's are independent and uniformly at random chosen from $\left\{0, 1, \ldots, \frac{n}{b} - 1\right\}$. Thus, (i) all I_j's are independent from each other; (ii) $\frac{n}{b}(j-1) + y_j + 1$ uniformly distributes in $\left\{\frac{n}{b}(j-1)+1, \frac{n}{b}(j-1)+2, \ldots, \frac{n}{b}\cdot j\right\}$ and hence $I_j = 1$ if and only if $\frac{n}{b}(j-1) + y_j + 1 \in S_j$. Then, by definition of k_j we have $\mathrm{E}\left[I_j\right] = k_j b/n$ for $j \in [b]$, and furthermore, $\mathrm{E}\left[\sum_{j=1}^{b} I_j\right] = \sum_{j=1}^{b} \mathrm{E}\left[I_j\right] = \sum_{j=1}^{b} \frac{k_j b}{n} = \frac{kb}{n}$.

Since $\mathsf{H}_\infty[Z_1] = \sum_{j=1}^{b} I_j$, $\mathrm{E}\left[\mathsf{H}_\infty[Z_1]\right] = \frac{kb}{n}$ and $I_1, \ldots, I_b$ are independent we upper bound $\Pr\left[\mathsf{H}_\infty[Z_1] < \alpha\right]$ by Hoeffding's inequality (see below)

$$
\begin{aligned}
&\Pr\left[\mathsf{H}_\infty[Z_1] < \alpha\right] \\
=&\Pr\left[\sum_{j=1}^{b} I_j < \alpha\right] = \Pr\left[\sum_{i=j}^{b} I_j < \mathrm{E}\left[\sum_{j=1}^{b} I_j\right] - \delta b\right] \\
\leq&\exp\left(-\frac{2(\delta b)^2}{b}\right) = \exp\left(-2\delta^2 b\right)
\end{aligned}
$$

Lemma 5 (Hoeffding's Inequality)**.** *For independent almost surely bounded random variables $X_1, \ldots, X_n$, i.e. $\Pr[X_i \in [a_i, b_i]] = 1$ for $i \in [n]$, let $Y = X_1 + \cdots + X_n$. Then, for any positive Δ,*

$$
\Pr\left[Y - \mathrm{E}\left[Y\right] \geq \Delta\right] \leq \exp\left(-\frac{2\Delta^2}{\sum_{i=1}^{n}(b_i - a_i)^2}\right)
$$

Since $\Pr\left[\mathsf{H}_\infty[Z_1] < \alpha\right] \leq \exp\left(-2\delta^2 b\right)$ and Z_1 is symmetric[3] to $Z_2, \ldots, Z_{n/b}$, by union bound he have that

$$
\Pr\left[\exists j \in [\frac{n}{b}] \text{ such that } \mathsf{H}_\infty[Z_j] < \alpha\right] \leq \frac{n}{b} \cdot \exp\left(-2\delta^2 b\right) = 2^{-\Omega(b)} \cdot \frac{n}{b}
$$

[2]Here, we abuse notation and we write Z instead of z because we wish to refer to the transformation of the algorithm on the statistical source. For the same reason we use $\overline{X}_j$ in the argument instead of $\overline{x}_j$ in the description of RRB to emphasis it as a random variable depending on X.

[3]Unlike other sources that lack such a degree of independence, bit-fixing sources are technically easier to handle exactly because the random bits are independent and this induces symmetry for all Z_i's.

Noticing that Z is a permutation of X and X is bit-fixing we have that $\mathsf{H}_\infty[Z_j \mid Z_1, Z_2, \ldots, Z_{j-1}] = \mathsf{H}_\infty[Z_j]$ for every $j \in [\frac{n}{b}]$. The above inequality is equivalent to

$$\Pr\left[\forall j \in [\frac{n}{b}],\ \mathsf{H}_\infty[Z_j \mid Z_1, \ldots, Z_{j-1}] \geq \alpha = \left(\frac{k}{n} - \delta\right) b\right] \geq 1 - \frac{n}{b} \cdot \exp\left(-2\delta^2 b\right)$$

□

We introduce the Leftover Hash Lemma [58, 59] and use it to prove Lemma 7, which asserts that a single extractor with the same seed suffices to extract randomness from each of the blocks.

Lemma 6 (Leftover Hash Lemma [58, 59])**.** *Let $S \subseteq \{0,1\}^n$ and $|S| \geq 2^\alpha$. Let $\rho > 0$ and $\mathcal{H}$ be a family of 2-universal hash functions mapping n bits to $\alpha - \rho$ bits. Then, the distribution $(h, h(x))$ is at most $2^{-\rho/2}$-close to uniform, when h is chosen uniformly at random from $\mathcal{H}$ and x uniformly from S.*

Lemma 7. (cf. Lemma 6 in [26]). *Let $\mathcal{H}$ be a family of 2-universal hash functions mapping b bits to $\alpha - \rho$ bits. If a random variable $Z = (Z_1, \ldots, Z_\ell)$ has next-block-min-entropy α and h is chosen uniformly at random from $\mathcal{H}$, then $(h, h(Z_1), \ldots, h(Z_\ell))$ is $2^{-\rho/2} \cdot \ell$ close to uniform distribution.*

Proof of Lemma 7. Let $H_i(Z) = \big(h, h(Z_1), \ldots, h(Z_i), \mathcal{U}_{(\alpha-\rho)(\ell-i)}\big)$, for $i \in \{0, 1, \ldots, \ell\}$. In particular, we have $H_0(Z) = \mathcal{U}_{|h|+(\alpha-\rho)\ell}$ and $H_\ell(Z) = (h, h(Z_1), \ldots, h(Z_\ell))$. Thus, it suffices to bound $\mathcal{SD}(H_\ell(Z), H_0(Z))$.

Since $Z = (Z_1, \ldots, Z_\ell)$ has next-block-min-entropy α it follows that

$$\mathsf{H}_\infty\left[Z_i \mid h(Z_1), \ldots, h(Z_{i-1})\right] \geq \mathsf{H}_\infty\left[Z_i \mid Z_1, \ldots, Z_{i-1}\right] \geq \alpha$$

Therefore, by the Leftover Hash Lemma [58, 59], $\mathcal{SD}(H_i(Z), H_{i-1}(Z)) \leq 2^{-\rho/2}$ holds for every $i \in \{1, \ldots, \ell\}$. Finally, we have

$$\mathcal{SD}(H_\ell(Z), H_0(Z)) \leq \sum_{i=1}^{\ell} \mathcal{SD}(H_i(Z), H_{i-1}(Z)) \leq 2^{-\rho/2} \cdot \ell$$

□

The 2-universal family of hash function in Lemma 7 can be uniformly sampled with $b + \alpha - \rho - 1 \leq 2b$ bits. For concreteness define $h : \{0,1\}^b \to \{0,1\}^{\alpha-\rho}$ as follows

$$h(\mathbf{z}) = \mathbf{T}\mathbf{z}$$

where $\mathbf{T}$ is a randomly chosen $b \times (\alpha - \rho)$ Toeplitz matrix, i.e. by uniformly sampling $b + \alpha - \rho - 1$ elements from $\mathsf{GF}[2]$, and all operations are over $\mathsf{GF}[2]$. Note that there exist 2-universal hash functions that can be sampled with b-many bits that is not practically realizable for big sources, this is why we use Toeplitz here.

By Lemma 4 and Lemma 7, and by setting $\ell = b_O = \frac{n}{b}$ we have that with probability at least $1 - \frac{n}{b} \cdot \exp\left(-2\delta^2 b\right)$ over the random choice of Y, $\mathcal{SD}\left(\mathsf{RRB}(X, Y), \mathcal{U}_{(\alpha-\rho)b_O}\right) \leq 2^{-\rho/2} \cdot \frac{n}{b}$. That is, for X and $Y \sim \mathcal{U}_d$ as above, and for $m = (\alpha - \rho)\ell = \left(\left(\frac{k}{n} - \delta\right) b - \rho\right) \frac{n}{b} = k - \delta n - \frac{\rho n}{b}$,

$$\mathcal{SD}\left(\mathsf{RRB}(X, Y), \mathcal{U}_{(\alpha-\rho)b_O}\right) \leq \left(2^{-\rho/2} + \exp\left(-2\delta^2 b\right)\right) \cdot \frac{n}{b}$$

Consequently, RRB is a (k, ε)-extractor for $k = \Omega(n)$, $\varepsilon = \left(2^{-\rho/2} + \exp\left(-2\delta^2 b\right)\right) \cdot \frac{n}{b}$ and $m = k - \left(\delta + \frac{\rho}{b}\right) n$. For every constant $\delta > 0$, it suffices to set $\rho = 2 \log \frac{n}{\varepsilon}$ and $b = \frac{1}{2\delta^2} \ln \frac{n}{\varepsilon} = \Theta(\log \frac{n}{\varepsilon})$, such that $m = k - (\delta + 4\delta^2 \log e)n$. Therefore, RRB is a (k, ε)-extractor for bit-fixing sources with $m = k - o(k)$ and $b = \Theta(\log \frac{n}{\varepsilon})$ as long as $k = \Omega(n)$.

Moreover, RRB is streaming computable with $\mathrm{O}\left(\log \frac{n}{\varepsilon}\right)$ memory and $\mathrm{O}(\log b) = \mathrm{O}\left(\log\log \frac{n}{\varepsilon}\right)$ passes over two streams by Claim 2. □

2.5 Streaming extraction from general weak sources with RRB extractor

In this section, we prove that for every (n,k)-source satisfying $k = \Omega(n)$, RRB works with $b = \mathrm{O}\left(\log\frac{n}{\varepsilon}\right)$, $b_O = \Omega\left(\frac{n}{b}\right)$, and $m = \Omega(n)$. More specifically, we show in Theorem 8 that Stages I and II of RRB provably achieve a strong next-block-min-entropy guarantee. Then, immediately by Lemma 7, Stage III extracts almost uniform random bits, as concluded in Corollary 9.

For simplicity, we let $n' = n/b$ and $Z = Z(X,Y)$ denote the content of σ_1 in step 5 (after Stage I and II) in the computation of $\mathsf{RRB}(X,Y)$. Recall the notation in Lemma 4, where Z is partitioned into $Z = (Z_1,\ldots,Z_{n'})$ such that each Z_i refers to a block. Then, the next-block-min-entropy guarantee is stated in the following theorem.

Theorem 8. *For every constant $\kappa > 0$ and every $(n,\kappa n)$-source X, for a uniform random seed Y, and for every sub-exponential error tolerance $\varepsilon = 2^{-o(n)}$, there exists $b = \mathrm{O}\left(\log\frac{n}{\varepsilon}\right)$, such that $Z(X,Y) = \left(Z_1,\ldots,Z_{n'}\right)$ is ε-close to a distribution $\widetilde{Z} = \left(\widetilde{Z}_1,\ldots,\widetilde{Z}_{n'}\right)$ where the first $\Omega(n')$, blocks in $\widetilde{Z}$ have next-block-min-entropy $\Omega(b)$.*

Corollary 9. *For sufficiently large b such that the distribution $\widetilde{Z}$ in Theorem 8 has next-block-min-entropy at least $b_e \geq 3\log\frac{n}{\varepsilon}$, we can extract $\Omega(n)$ bits that are 2ε-close to uniform, by paying another $\mathrm{O}(b)$ random bits for the hash function $h : \{0,1\}^b \to \{0,1\}^{b_e - 2\log\frac{n}{\varepsilon}}$ applied on those first $\Omega(n')$ blocks of $\widetilde{Z}$.*

This section is organized as follows. We first explain in Section 2.5.1 the proof intuition of Theorem 8 based on a matrix view of RRB computation. Then, we formalized the definition of goodness mentioned in the proof intuition and introduce important lemmas in Section 2.5.2 (some of the proofs are deferred to Section 2.5.4 and 2.5.5). The full proof of Theorem 8 is given in Section 2.5.3.

2.5.1 Proof intuition of Theorem 8

Before presenting the proof intuition, we introduce the matrix view of the computation of RRB. This view significantly simplifies the exposition, but we do not look at this matrix as an operator.

First, we denote the n-bit long input by $X = \left(X_{1,1}, X_{1,2}\ldots, X_{1,n'}, X_{2,1},\ldots,X_{2,n'},\ldots,X_{b,1},\ldots,X_{b,n'}\right)$, and let X be represented as a $b \times n'$ matrix

$$X = \begin{pmatrix} X_{1,1} & \cdots & X_{1,n'} \\ X_{2,1} & \cdots & X_{2,n'} \\ \vdots & \vdots & \vdots \\ X_{b,1} & \cdots & X_{b,n'} \end{pmatrix}$$

Then, let $\overline{X}_i = \left(X_{i,1},\ldots,X_{i,n'}\right)$ denote the i-th row in the above matrix of X, and $\left(Y_1,\ldots,Y_b\right)$ be the part of Y used for random re-bucketing, where $Y_i \in \mathbb{Z}_{n'} = \{1,2,\ldots,n'\}$ and the addition is modular n', i.e. $Y_i + n' = Y_i$. Now, the cyclic shift of X with seed with the vector Y becomes

$$\mathsf{shift}(X,Y) = \begin{pmatrix} \mathsf{shift}(\overline{X}_1, Y_1) \\ \mathsf{shift}(\overline{X}_2, Y_2) \\ \vdots \\ \mathsf{shift}(\overline{X}_b, Y_b) \end{pmatrix} = \begin{pmatrix} X_{1,Y_1+1} & \cdots & X_{1,n'} & X_{1,1} & \ldots & X_{1,Y_1} \\ X_{2,Y_2+1} & \cdots & & & \cdots & X_{2,Y_2} \\ \vdots & \vdots & & & & \vdots \\ X_{b,Y_b+1} & \cdots & & & \cdots & X_{b,Y_b} \end{pmatrix} \tag{1}$$

Recall that Z is the re-bucketing of the shifted blocks $\mathsf{shift}(X,Y)$, and hence

$$\begin{aligned} Z(X,Y) &= (Z_1,\ldots,Z_j,\ldots,Z_b) \\ &= \Big(\big(X_{1,Y_1+1},\ldots,X_{b,Y_b+1}\big),\ldots,\big(X_{1,Y_1+j},\ldots,X_{b,Y_b+j}\big),\ldots,\big(X_{n',Y_1+n'},\ldots,X_{b,Y_b+n'}\big)\Big) \end{aligned} \tag{2}$$

Notice that for every $j \in [n']$, the block Z_j in (2) corresponds to the j-th column in the matrix representation of $\mathsf{shift}(X,Y)$ in (1). For every $j \in [n']$, we denote $Z_j = (Z_{1,j}, \ldots, Z_{b,j})^T$ as a column vector where $Z_{i,j} = Z(X,Y)_{i,j} = X_{i,Y_i+j}$. Thus, we get the matrix representation of Z as follows.

$$Z(X,Y) = \begin{pmatrix} Z_1 & \cdots & Z_{n'} \end{pmatrix} = \begin{pmatrix} Z_{1,1} & \cdots & Z_{1,n'} \\ \vdots & \vdots & \vdots \\ Z_{b,1} & \cdots & Z_{b,n'} \end{pmatrix} = \mathsf{shift}(X,Y) \tag{3}$$

Note that, we think of the input X as a collection of rows, whereas Z as a collection of columns.

Proof intuition of Theorem 8. The goal of this theorem is to show the $\Omega(b)$ next-block-min-entropy guarantee for the first blocks of $\widetilde{Z}$. By definition, the next-block-min-entropy guarantee of a single block $\widetilde{Z}_j$ in $\widetilde{Z}$ requires $\Pr\left[\widetilde{Z}_j = z_j \middle| \underbrace{\widetilde{Z}_1 = z_1, \ldots, \widetilde{Z}_{j-1} = z_{j-1}}_{\mathcal{E}}\right] \le 2^{-\Omega(b)}$ for every choice of strings $z_1, \ldots, z_j \in \{0,1\}^b$. For simplicity, we let $\mathcal{E} = \mathcal{E}(z_1, \ldots, z_j)$ denote the event that the first $j-1$ blocks in $\widetilde{Z}$ follows the specific choice of strings, i.e. $\widetilde{Z}_1 = z_1, \ldots, \widetilde{Z}_{j-1} = z_{j-1}$.

Then, we apply the chain rule to decompose the conditional probability of $\Pr\left[\widetilde{Z}_j = z_j \middle| \mathcal{E}\right]$ as follows

$$\Pr\left[\widetilde{Z}_j = z_j \middle| \mathcal{E}\right] = \prod_{i=1}^{b} \Pr\left[\widetilde{Z}_{i,j} = z_{i,j} \middle| \mathcal{E},\ \widetilde{Z}_{1,j} = z_{1,j}, \ldots, \widetilde{Z}_{i-1,j} = z_{i-1,j}\right] \tag{4}$$

As a result, in order to upper bound $\Pr\left[\widetilde{Z}_j = z_j \middle| \mathcal{E}\right]$, it suffices to show that $\Omega(b)$ many terms in the product are no more than $1/2$.

Whenever $\Pr\left[\widetilde{Z}_{i,j} = z_{i,j} \middle| \widetilde{Z}_1 = z_1, \ldots, \widetilde{Z}_{j-1} = z_{j-1},\ \widetilde{Z}_{1,j} = z_{1,j}, \ldots, \widetilde{Z}_{i-1,j} = z_{i-1,j}\right] \le 1/2$, we say that $z_{i,j}$ is "good" for $\widetilde{Z}_j = z_j$, since this $z_{i,j}$ will contribute a factor of $1/2$ to the upper bound of $\Pr\left[\widetilde{Z}_j = z_j \middle| \mathcal{E}\right]$. Now, it suffices to show that there are $\Omega(b)$ many "good" bits for $\widetilde{Z}_j = z_j$. Intuitively, every such good bit contributes at least one bit information to $\widetilde{Z}_j = z_j$ conditioned on previous blocks/bits. Note, some care is needed to develop an appropriate order that induces a useful notion of "previous" in our formal definition of goodness. In fact, this is a subtle issue that we develop progressively in Section 2.5.2.

Finally, the number of "good bits" in a single block Z_j, which is statistically close to $\widetilde{Z}_j$, can be lower bounded through a novel application of the standard concentration bound over the random choice of Y. The concentration bound will be applicable because: (1) every bit $Z_{i,j} = \mathsf{shift}(\overline{X}_i, Y_i)_j$ in the j-th block Z_j is determined by a cyclic shift of $\overline{X}_i$ with independently selected sub-seed Y_i; and more importantly, (2) the independence of Y_i's is translated to the independence of every bit in Z_j being good under our appropriate definition of goodness, although this definition inevitably involves sub-seeds other than Y_i.

2.5.2 A useful definition of goodness and important lemmas

in this part, we formalize the notion of "goodness", analyze its property, and introduce important lemmas.

A useful definition of "good". As discussed in the proof intuition, $z_{i,j}$ is "good" if $Z_{i,j} = z_{i,j}$ with probability at most $1/2$ conditioned on the event that Z coincides a fixed string z on every bit preceding $Z_{i,j}$ under an appropriate order. Now, let us specify this order for the formal definition of "goodness". To realize a stronger form of next-block-min-entropy (stronger because we condition on even more bits) and apply the concentration bound, this specific order should ideally satisfy the following three conditions.

(a) The preceding bits of different bits in Z_j, i.e. $Z_{1,j}, Z_{2,j}, \ldots, Z_{b,j}$, increase in a monotone way. That is, for every $1 \le i_1 < i_2 \le b$, the set of bits preceding $Z_{i_1,j}$ is a proper subset of that of $Z_{i_2,j}$.

(b) $Z_{i,j}$ appears after all the $b(j-1)$ many bits in the first $j-1$ blocks, i.e. $Z_1, \ldots, Z_{j-1}$.

(c) Preceding bits of $Z_{i,j}$ are independent from any sub-seed $Y_{i'}$ as long as $i' \neq i$.

Condition (a) is indispensable for applying the chain rule as in (4). Condition (b) is necessary for analyzing next-block-min-entropy of Z_j, which is defined conditioning on the first $j-1$ blocks. Condition (c) guarantees the event that "$Z_{i,j}$ is good" independent from the choice of $Y_{i'}$, which provides us with useful independence required by the concentration bounds. The latter will show that Z_j contains sufficiently many "good bits" with overwhelmingly high probability.

We will see that we cannot satisfy this ideal scenario of all of the above conditions. Every total order would satisfy Condition (a). The natural order of Z (i.e. column-by-column as in (2)) satisfies Condition (b) but not (c), whereas the naive row-by-row order satisfies (c) but not (b). In fact, Conditions (b) and (c) are inherently inconsistent. As a compromise, we have to relax Condition (b) by fixing $j = j_0$ and Condition (c) by adding $i' > i$ (instead of $i' \neq i$). The compromised conditions can be simultaneously fulfilled by the following (Y, j_0)-order.

Definition 10. *For every $j_0 \in \{1, 2, \ldots, n'\}$ and for every $y = (y_1, \ldots, y_b) \in \{0,1\}^d = (\{0,1\}^{\log n'})^b$, the (y, j_0)-*order *is a sequence of Z-indices as follows:*

$$\begin{aligned}
&(1,1), (1,2), \ldots, (1, j_0-1), (2,1), \ldots, (2, j_0-1), \ldots, (b,1), \ldots, (b, j_0-1),\\
&(1, n'-y_1+1), \ldots, (1, n'),\ (1, j_0),\ (1, j_0+1), \ldots, (1, n'-y_1),\\
&\ldots,\\
&(i, n'-y_i+1), \ldots, (i, n'),\ (i, j_0),\ (i, j_0+1), \ldots, (i, n'-y_i),\\
&(i+1, n'-y_{i+1}+1), \ldots, (i+1, n'),\ (i+1, j_0),\ (i+1, j_0+1), \ldots, (i+1, n'-y_{i+1}),\\
&\ldots,\\
&(b, n'-y_b+1), \ldots, (b, n'),\ (b, j_0),\ (b, j_0+1), \ldots, (b, n'-y_b)
\end{aligned}$$

In case $y_i \in \{n', n'-1, \ldots, n'-j_0+1\}$, the $(i+1)$-th line is set to $(i, j_0), (i, j_0+1), \ldots, (i, n')$. This solves the ambiguity that the index $(i, n'-y_i+1)$ may be counted twice when $n'-y_i+1 \le j_0$.

*Whenever j_0 is clear from context, we call it a y-*order *for short. Let $\overline{y}_i = (y_1, y_2, \ldots, y_i)$ denote the first i elements in y, then the $\overline{y}_i$-*order *is defined as a prefix of the above sequence up to the term $(i, n'-y_i)$, i.e. the first $i+1$ lines which are determined by $\overline{y}_i$.*

For every given seed $y \in \{0,1\}^d$, the length $n = b \times n'$ string z can be represented in y-order as $(z)_y = \mathsf{w}_0 z_{1,j_0} \mathsf{w}_1 z_{2,j_0} \mathsf{w}_2 z_{3,j_0} \ldots \mathsf{w}_{i-1} z_{i,j_0} \mathsf{w}_i \ldots z_{b,j_0} \mathsf{w}_b$, where each w_i denotes a sub-sequence of z restricted on indices between (i, j_0) and $(i+1, j_0)$ as in the y-order, for every $0 \le i \le b$.

A visual depiction of the (y, j_0)-order is given in Figure S1.

Then, we introduce the definition of j_0-goodness, which is defined for the specific j_0 and given (x, y). Roughly speaking, an X-index (i, j) is called j_0-*good in* (x, y), if the conditional probability of $X_{i,j} = x_{i,j}$ is less than $1/2$ when $X_{i,j}$ shifted to Z_{i,j_0} and the rest part of Z that precedes Z_{i,j_0} are compatable with (x, y).

Definition 11. *For every $j_0 \in \{1, 2, \ldots, n'\}$, and for every string $x \in \{0,1\}^n$ and seed $y \in \{0,1\}^d$, we let $z = Z(x, y)$ as in (2), and define the j_0-*goodness *of an X-index (i, j) regarding (x, y) for every $(i, j) \in [b] \times [n']$*

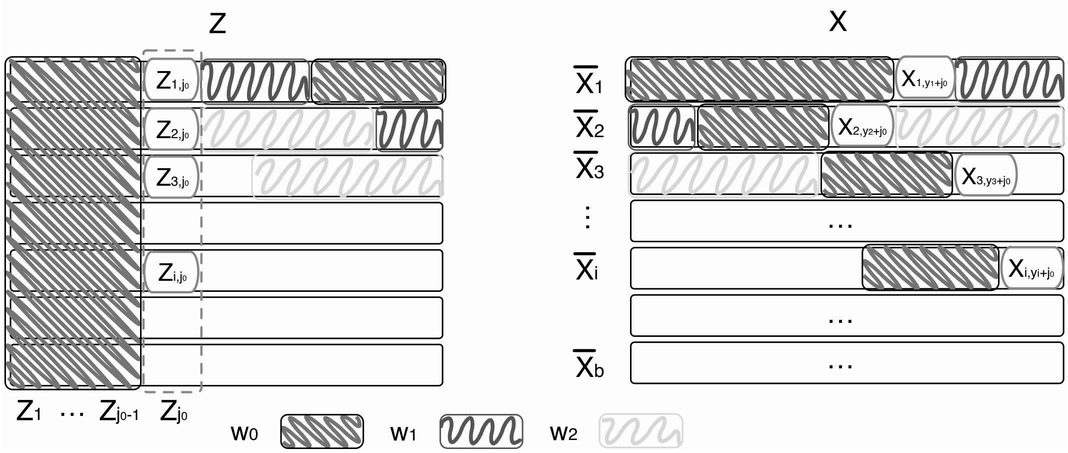

Figure S1: Since Z is essentially a permutation of X, we describe the (y, j_0)-order in terms of both matrices of Z and X. The gray part w_0 consists of the first $j_0 - 1$ columns of Z and all the bits preceding X_{1,y_1+j_0} (which is shifted to Z_{1,j_0} when $Y_1 = y_1$) in $\overline{X}_1$. For $i \geq 1$, the sub-string w_i is defined following the natural row-by-row order in X except for those bits already covered by w_0.

as follows

$$\begin{aligned} p_{i,j}^{j_0}(x,y) = \Pr_{X\sim\mathcal{D},Y\sim\mathcal{U}_d}\big[X_{i,j}=x_{i,j}\big| \quad & \underbrace{X_{1,1}=x_{1,1}, X_{1,2}=x_{1,2}, \ldots, X_{i-1,n'}=x_{i-1,n'}}_{\mathcal{E}_1(i-1,x)}, \\ & \underbrace{Z_{i+1,1}=z_{i+1,1}, Z_{i+1,2}=z_{i+1,2}, \ldots, Z_{b,j_0-1}=z_{b,j_0-1}}_{\mathcal{E}_2(i+1,j_0-1,x,y)}, \\ & \underbrace{X_{i,1}=x_{i,1}, X_{i,2}=x_{i,2}, \ldots, X_{i,j-1}=x_{i,j-1}}_{\mathcal{E}_3(i,j-1,x_i)}\big] \\ = \Pr_{X\sim\mathcal{D},Y\sim\mathcal{U}_d}\big[X_{i,j}=x_{i,j}\big| \quad & \mathcal{E}_1(i-1,x),\ \mathcal{E}_2(i+1,j_0-1,x,y),\ \mathcal{E}_3(i,j-1,x_i)\big] \end{aligned}$$

The X-index (i,j) is called "good for the prefix of x up to (i,j) under the (y,j_0)-order"*, or simply* "j_0-good in (x,y)"*, if either $p_{i,j}^{j_0}(x,y) < 1/2$, or $p_{i,j}^{j_0}(x,y) = 1/2$ but $x_{i,j} = 0$. Otherwise, the X-index (i,j) is called* j_0-bad in (x,y). *Whenever (x,y) and j_0 are clear from context, we also say that (i,j) is* good *(resp.* bad*) for short instead of j_0-good in (x,y) (resp. j_0-bad in (x,y)).*

Recalling that Z is essentially a permutation of X specified by Y where in particular $Z_{i,j} = X_{i,j+Y_i}$, we say that a Z-index (i,j) is j_0-good in (x,y) *if the X-index $(i, j+y_i)$ is j_0-good in (x,y), where y_i is the i-th sub-seed of y that specifies the cyclic shift of $\overline{X}_i$.*

The additional requirement of $x_{i,j} = 0$ for the case $p_{i,j}^{j_0}(x,y) = 1/2$ is to ensure that exactly one of $x_{i,j} = 0$ and $x_{i,j} = 1$ makes the index j_0-good for any given prefix under the (y,j_0)-order. This provides the injectivity used in Lemma 15.

Now, we discuss the meanings of $\mathcal{E}_1(i-1,x), \mathcal{E}_2(i+1,j_0-1,x,y), \mathcal{E}_3(i,j-1,x_i)$ in the above definition of $p_{i,j}^{j_0}(x,y)$ and how they make this definition useful. A visual description of their ranges is given in Figure S2.

The first event $\mathcal{E}_1(i-1,x)$ quantifies exactly the first $i-1$ rows in X-indices and hence it is independent from Y. On the other hand, $\mathcal{E}_1$ contains all the information of the first $i-1$ rows in the Z-matrix when Y is given. In particular, if $\overline{Y}_{i-1} = \overline{y}_{i-1}$, $\mathcal{E}_1(i-1,x)$ is equivalent to Z coinciding z in the first $i-1$ rows.

The second event $\mathcal{E}_2(i+1,j_0-1,x,y)$ quantifies the $(b-i)\times(j_0-1)$ left-bottom part of the Z-matrix, which is an indispensable part of $(Z_1,\ldots,Z_{j_0-1})$.

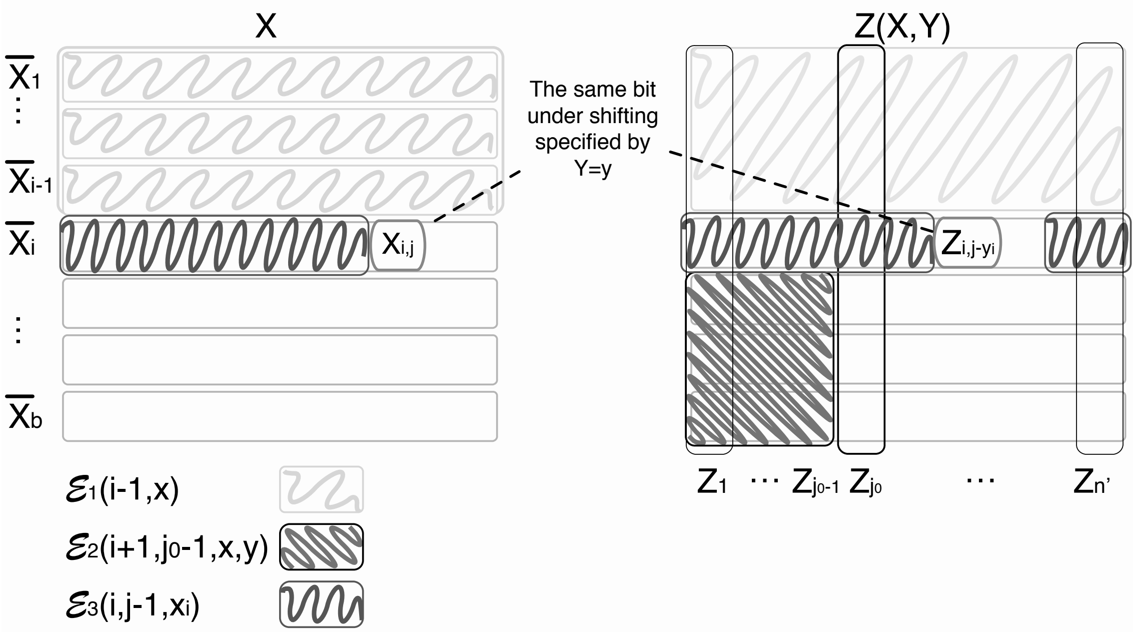

Figure S2: The involved ranges of the conditioning events in $p_{i,j}^{j_0}(x, y)$. The bits quantified in $\mathcal{E}_1, \mathcal{E}_2$ and $\mathcal{E}_3$ are depicted respectively in green, gray, and blue. When $Y_i = y_i$, $X_{i,j}$ (the red cell) is shifted to $Z_{i,j-y_i}$, while the blue part referring to $\mathcal{E}_3(i, j-1, x_i)$ is cyclicly shifted as well.

The last event $\mathcal{E}_3(i, j-1, x_i)$ is the most subtle one. It asserts that the length-$(j-1)$ prefix of $\overline{X}_i$ coincides to that of x, which covers the necessary part of $(Z_1, \dots, Z_{j_0-1})$ when $j \geq j_0$. This specific design of $\mathcal{E}_3$ enjoys the following advantage: (i) the sub-seed y_i cannot influence the j_0-goodness of any X-index (i, j), whereas, (ii) it is able to affect the j_0-goodness of every Z-index (i, j), which corresponds to X-index $(i, j + y_i)$. More specifically, the j_0-good X-indices in the i-th row are independent from y_i, whereas the j_0-good Z-indices in the i-th row are shifted with an offset y_i.

The only drawback of $\mathcal{E}_3(i, j-1, x_i)$ is that it does not necessarily cover the first $j_0 - 1$ bits (i.e. $Z_{i,1}, \dots, Z_{i,j_0-1}$) in the i-th row of Z. To that end, we introduce the notion of j_0-*super-good*, which eliminates j_0-good X-indices (i, j) with $j < j_0$. For simplicity in the argument, we further pick an upper bound ℓ (typically $\ell = \Omega(n')$) and in each row we only keep the last ℓ many j_0-super-good X-indices.

Definition 12. *For every $j_0 \in \{1, 2, \dots, n'\}$ and $(x, y) \in \{0,1\}^n \times \{0,1\}^d$, we say that an X-index (i, j) is* j_0-super-good in (x, y) *if $j \in \{j_0, j_0 + 1, \dots, n'\}$ and (i, j) is already j_0-good in (x, y). The j_0-super-goodness of a Z-index (i, j) is the same as the X-index $(i, y_i + j)$. Furthermore, for $\ell \in [n']$, a j_0-super-good-in-(x, y) X-index (i, j) is called* (j_0, ℓ)-good in (x, y) *if $|\{(i, j+1), \dots, (i, n')\} \bigcap \{j_0\text{-super-good indices in } \overline{X}_i\}| \leq \ell - 1$. Similarly, (j_0, ℓ)-goodness of a Z-index (i, j) follows that of the X-index $(i, y_i + j)$.*

These two "enhanced" notions of goodness differ from j_0-goodness only in $\mathcal{E}_3$. However, the changes are independent of y_i, and therefore the advantage of $\mathcal{E}_3$ is preserved: y_i cannot determine whether an X-index (i, j) is good (i.e. j_0-super-good or (j_0, ℓ)-good), but it matters for every Z-index (i, j). We summarize the properties of all these events in the following remark.

Remark 13. *In the i-th row of Z-matrix, the* number *of Z-indices that are j_0-good (resp. j_0-super-good or (j_0, ℓ)-good) in (x, y) is fully determined by x and $y_{i+1}, \dots, y_b$.*

For fixed x, $y_{i+1}, \dots, y_b$ and uniformly selected y_i, and for every $j \in \{1, \dots, n'\}$, the Z-index (i, j) is j_0-good (resp. j_0-super-good or (j_0, ℓ)-good) in (x, y) with equal probability. For example (similar for (i, j_0) being

j_0-super-good and (j_0,ℓ)-good),

$$\Pr_{y_i \leftarrow \mathcal{U}_{[n']}}\left[Z\text{-index } (i,j_0) \text{ is } j_0\text{-good in } (x,y)\right] = \left|\{j_0\text{-good-in-}(x,y) \text{ indices in the } i\text{-th row of } X\}\right| / n'$$

It is easy to verify that Definition 12 fulfills Conditions (a), (b), and (c) on page 15 for a useful definition of goodness, though only in the relaxed version where $j = j_0$ and $i' > i$. Luckily, this is already sufficient for our proof. Since Definition 12 is induced from Definition 11, we begin the argument with j_0-goodness and later on turn to (j_0,ℓ)-goodness when necessary.

Good blocks and useful lemmas. The definition of a good index quantifies merely a single bit, whereas our goal is to lower bound the next-block-min-entropy of a whole block Z_{j_0}. Therefore, we introduce the following notion of informative and good blocks, which allows us to formally talk about the number of good indices in the whole matrix and in a single block respectively.

Definition 14. *For every $\alpha > 0$ and every $j_0 \in \{1,\dots,n'\}$, the combination of input string and seed $(x,y) \in \{0,1\}^n \times \{0,1\}^d$ is called* α-informative for j_0 *if there are at least αn many Z-indices (or equivalently, X-indices) being j_0-good in (x,y). Furthermore, we say that a single string $x \in \{0,1\}^n$ is* α-informative (for j_0) *if for every $y \in \{0,1\}^d$, (x,y) is α-informative for j_0.*

Then, we lower bound the probability that a randomly chosen x is α-informative as follows.

Lemma 15. *For every constant $\kappa > 0$ and for every positive integer $j_0 \le 1 + \kappa n/(2b)$, there is a constant $c_1 > 0$ such that for every $(n,\kappa n)$-source X and for arbitrarily fixed seed $y \in \{0,1\}^d$,*

$$\Pr_{X\sim\mathcal{D}}\left[(X,y) \text{ is not } \alpha\text{-informative for } j_0\right] \le 2^{-c_1\kappa n} = 2^{-\Omega(n)}$$

where $\alpha = c_1\kappa/\log\kappa^{-1} = \Theta(1)$.

Proof. Since j_0 and y are fixed, we let $x \sim X$ and realize the binary matrix $Z(x,y)$ as a function of x. The matrix representation of $z = Z(x,y) \in \{0,1\}^{b\times n'}$ can be mapped to matrices in $\{\text{good,bad}\}^{b\times n'}$ where the (i,j)-th entry of the matrix is good whenever the Z-index (i,j) is j_0-good in (x,y), and bad otherwise. Moreover, when fixing the $b \times (j_0-1)$ many bits in first j_0-1 columns of z, the rest part of z is injectively mapped to the good/bad matrices.

Recall that for the fixed seed y, the binary matrix z is just a fixed permutation of x. Thus, when we fix x in $(j_0-1)b$ many bits that corresponds to the first j_0-1 columns of z, the map from the rest part of x to matrices in $\{\text{good,bad}\}^{b\times n'}$ is also injective. For different choices of these $(j_0-1)b$ bits, at most $2^{(j_0-1)b}$ many distinct strings x's can be mapped to the same good/bad matrix.

Now, we consider the map from the string $x \in \{0,1\}^n = \{0,1\}^{b\times n'}$ to good/bad matrices in $\{\text{good,bad}\}^{b\times n'}$. On one hand, a string x is not α-informative only when there exists $y \in \{0,1\}^d$ such that given this unfavorable y, the string x is mapped to a good/bad matrix with less than αn many "good" entries. The number of such good/bad matrices is $\sum_{i=0}^{\lceil\alpha n\rceil-1}\binom{n}{i}$. On the other hand, every such matrix has at most $2^{(j_0-1)b}$ many distinct pre-images of x's for the fixed y. As a result, the number of distinct strings x's such that (x,y) is not α-informative for j_0 is upper bounded as below

$$\left|\left\{x \in \{0,1\}^n \;\middle|\; (x,y) \text{ is not } \alpha\text{-informative for } j_0\right\}\right| \le 2^{(j_0-1)b}\sum_{i=0}^{\lceil\alpha n\rceil-1}\binom{n}{i}$$

Since X is an $(n,\kappa n)$-source, each string $x \in \{0,1\}^n$ appears in X with probability at most $2^{-\kappa n}$. Therefore, when $(j_0-1)b \le \kappa n/2$ and $\alpha = c_1\kappa/\log\kappa^{-1}$,

$$\Pr\left[(X,y) \text{ is not } \alpha\text{-informative for } j_0\right] \le 2^{-\kappa n} \times 2^{(j_0-1)b}\sum_{i=0}^{\lceil\alpha n\rceil-1}\binom{n}{i} \le 2^{-\kappa n/2}\sum_{i=0}^{\lceil\alpha n\rceil-1}\binom{n}{i} \le 2^{-c_1\kappa n}$$

when the positive constant c_1 is small enough. For instane, $c_1 \leq \kappa/6$ is sufficient for $\kappa \leq 1/2$. □

For the j_0-th block Z_{j_0} in Z, we introduce the following definition of a good block and the lemma showing that for every α-informative x, Z_{j_0} is a good block with high probability. The proof of Lemma 17 is deferred to Section 2.5.4 on page 21.

Definition 16. *For every $\alpha > 0$ and $j_0, \ell \in \{1, \ldots, n'\}$, and for every $(x, y) \in \{0,1\}^n \times \{0,1\}^d$, the block Z_{j_0} of Z, i.e. $Z_{j_0} = (Z_{1,j_0}, \ldots, Z_{b,j_0})$, is called an (α, j_0, ℓ)-*good block in (x, y) *if there are at least αb many indices in Z_{j_0} being (j_0, ℓ)-good in (x, y). For every $\beta > 0$, the block Z_{j_0} is called (α, j_0, ℓ)-*good within β *if*

$$\Pr_{X \sim \mathcal{D}, Y \sim \mathcal{U}_d} [Z_{j_0} \textit{ is } (\alpha, j_0, \ell)\textit{-good block in } (X, Y)] \geq 1 - \beta$$

Lemma 17. *For any constant $\alpha > 0$ and integer $j_0 \leq \alpha n'/4$, and for every $x \in \{0,1\}^n$ that is α-informative for j_0, there is a constant $\alpha' > 0$ and $\beta = 2^{-\Omega(b)}$ such that*

$$\Pr_{Y \sim \mathcal{U}_d} \left[Z_{j_0} \textit{ is an } (\alpha', j_0, \ell)\textit{-good block in } (x, Y)\right] \geq 1 - \beta$$

where $\ell = \lceil \alpha n'/2 \rceil - j_0 \geq \alpha' n/4 = \Omega(n')$.

Finally, we establish the relation from (α, j_0, ℓ)-good blocks to next-block-min-entropy.

Lemma 18. *If there is a constant $\alpha' > 0$ such that the block Z_{j_0} is (α', j_0, ℓ)-good, then*

$$\mathsf{H}_\infty \left[Z_{j_0} \middle| Z_1, \ldots, Z_{j_0 - 1}\right] \geq \alpha' b = \Omega(b)$$

If Z_j is (α', j, ℓ)-good for every $j = 1, 2, \ldots, j_0$, then $(Z_1, \ldots, Z_{j_0})$ has next-block-min-entropy $\Omega(b)$ in every block.

Note that this lemma requires Z_{j_0} to be (α', j_0, ℓ)-good, which we have to approximate within some small distance. The proof of Lemma 18 is given in Section 2.5.5 on page 23.

2.5.3 The RRB next-block-min-entropy guarantee

Theorem 8 (restated). For every constant $\kappa > 0$ and every $(n, \kappa n)$-source X, for a uniform random seed Y, and for every sub-exponential error tolerance $\varepsilon = 2^{-o(n)}$, there exists $b = O\left(\log \frac{n}{\varepsilon}\right)$, such that $Z(X, Y) = \left(Z_1, \ldots, Z_{n'}\right)$ is ε-close to a distribution $\widetilde{Z} = \left(\widetilde{Z}_1, \ldots, \widetilde{Z}_{n'}\right)$ where the first $\Omega(n')$, blocks in $\widetilde{Z}$ have next-block-min-entropy $\Omega(b)$.

Proof of Theorem 8. Combining Lemma 15 and Lemma 17, there is a constant $\alpha > 0$ such that for every $j_0 \leq \alpha n'/4$, the block Z_{j_0} is (α, j_0, ℓ)-good within $\beta' = 2^{-\Omega(b)}$, since

$$\begin{aligned}
&\Pr_{X \sim \mathcal{D}, Y \sim \mathcal{U}_d} \left[Z_{j_0} \text{ is } (\alpha', j_0, \ell)\text{-good in } (X, Y)\right] \\
&\geq \Pr_{X \sim \mathcal{D}} [X \text{ is } \alpha\text{-informative for } j_0] \Pr_{Y \sim \mathcal{U}_d} \left[Z_{j_0} \text{ is } (\alpha', j_0, \ell)\text{-good in } (X, Y) \middle| X \text{ is } \alpha\text{-informative}\right] \\
&\geq (1 - 2^{-\Omega(n)})(1 - \beta) \geq 1 - \beta'
\end{aligned}$$

where $\beta = 2^{-\Omega(b)}$ as in Lemma 17.

Set $j_0 = \alpha n'/4$ and apply the union bound,

$$\Pr_{X \sim \mathcal{D}, Y \sim \mathcal{U}_d} \left[\text{for every } 1 \leq j \leq j_0,\ Z_j \text{ is } (\alpha', j, \ell)\text{-good in } (X, Y)\right] \geq 1 - j_0 \beta'$$

Let the distribution of $(\widetilde{X},\widetilde{Y})$ be the conditional distribution of $(X,Y) \sim (\mathcal{D},\mathcal{U}_d)$ of the above event, i.e. $(\widetilde{X},\widetilde{Y}) \sim (X,Y)\big|_{\forall 1 \le j \le j_0,\ Z_j \text{ is } (\alpha', j, \ell)\text{-good in } (X,Y)}$. The new distribution $(\widetilde{X},\widetilde{Y})$ is $j_0\beta'$-close to (X,Y), and $\Pr_{\widetilde{X},\widetilde{Y}}\left[Z_j \text{ is } (\alpha', j, \ell)\text{-good in } (\widetilde{X},\widetilde{Y})\right] = 1$ for every $1 \le j \le j_0$.

Then, we let $\widetilde{Z} = Z(\widetilde{X},\widetilde{Y})$, and respectively $\widetilde{Z}_j = Z(\widetilde{X},\widetilde{Y})_j$ for every $j = 1, 2, \ldots, n'$. It immediately follows that $\widetilde{Z}_j = Z(\widetilde{X},\widetilde{Y})_j$ is $j_0\beta'$-close to $Z_j = Z(X,Y)_j$ by information processing lemma. Recalling that $\beta' = 2^{-\Omega(b)}$ and $j_0 < n' = n/b$, for every $z_{j_0} \in \{0,1\}^b$ and $\mathsf{w}_0 \in \{0,1\}^{b(j_0-1)}$, we apply Lemma 18 and get the following inequality.

$$\begin{aligned}
&\Pr_{\widetilde{X},\widetilde{Y}}\left[\widetilde{Z}_{j_0} = z_{j_0} \middle| (\widetilde{Z}_1, \ldots, \widetilde{Z}_{j_0-1}) = \mathsf{w}_0\right] \\
=& \Pr_{\widetilde{X},\widetilde{Y}}\left[Z_{j_0}(\widetilde{X},\widetilde{Y}) = z_{j_0} \middle| Z_{j_0} \text{ is } (\alpha', j_0, \ell)\text{-good in } (\widetilde{X},\widetilde{Y}) \text{ and } (\widetilde{Z}_1, \ldots, \widetilde{Z}_{j_0-1}) = \mathsf{w}_0\right] \\
\le& \Pr_{X,Y}\left[Z_{j_0}(X,Y) = z_{j_0} \middle| Z_{j_0} \text{ is } (\alpha', j_0, \ell)\text{-good in } (X,Y) \text{ and } (Z_1, \ldots, Z_{j_0-1}) = \mathsf{w}_0\right] + j_0\beta' \\
\le& 2^{-\alpha' b} + j_0\beta' \le n' 2^{-\Omega(b)} = 2^{\log(n/b) - \Omega(b)} = 2^{\log n - \Omega(b)}
\end{aligned}$$

For sufficiently large $b = \mathrm{O}\left(\log \frac{n}{\varepsilon}\right)$, the last $\Omega(b)$ in the above inequality becomes asymptotically larger than $\log n$. Therefore, we simplify it as

$$\Pr_{\widetilde{X},\widetilde{Y}}\left[\widetilde{Z}_{j_0} = z_{j_0} \middle| (\widetilde{Z}_1, \ldots, \widetilde{Z}_{j_0-1}) = \mathsf{w}_0\right] \le 2^{-\Omega(b)}$$

Since the conditional probability is bounded for arbitrary z_{j_0} and w_0, by definition of next-block-min-entropy we have

$$\mathsf{H}_\infty\left[\widetilde{Z}_{j_0} \middle| \widetilde{Z}_1, \ldots, \widetilde{Z}_{j_0-1}\right] \ge \Omega(b)$$

Note that exactly the same argument also works for $j < j_0$. Therefore,

$$\mathsf{H}_\infty\left[\widetilde{Z}_{j} \middle| \widetilde{Z}_1, \ldots, \widetilde{Z}_{j-1}\right] \ge \Omega(b)$$

for every $j \in \{1, \ldots, j_0\}$, where $j_0 = \alpha n'/4 = \Omega(n')$ and $b = \mathrm{O}\left(\log \frac{n}{\varepsilon}\right)$. Moreover, the distribution $\widetilde{Z}$ is ε-close to Z since $(\widetilde{X},\widetilde{Y})$ is $j_0\beta'$-close to (X,Y), and $j_0\beta' \le n' 2^{-\Omega(b)} \le \varepsilon$ for sufficiently large $b = \mathrm{O}\left(\log \frac{n}{\varepsilon}\right)$. Thus we complete the proof of Theorem 8. □

2.5.4 Proof of Lemma 17 – from min-entropy to the good block guarantee

Lemma 17 (restated). For any constant $\alpha > 0$ and integer $j_0 \le \alpha n'/4$, and for every $x \in \{0,1\}^n$ that is α-informative for j_0, there is a constant $\alpha' > 0$ and $\beta = 2^{-\Omega(b)}$ such that

$$\Pr_{Y \sim \mathcal{U}_d}\left[Z_{j_0} \text{ is an } (\alpha', j_0, \ell)\text{-good block in } (x, Y)\right] \ge 1 - \beta$$

where $\ell = \lceil \alpha n'/2 \rceil - j_0 \ge \alpha' n/4 = \Omega(n')$.

Proof. We fix an arbitrary $x \in \{0,1\}^n = \{0,1\}^{b \times n'}$ that is α-informative for j_0. Then, for every $y \in \{0,1\}^d$ there are at least $\alpha n = \alpha b n'$ many Z-indices being j_0-good in (x,y). In particular, there are at least $\lceil \alpha b/2 \rceil$ many "*good rows*" in the Z-matrix, where each "good row" contains at least $\lceil \alpha n'/2 \rceil$ many j_0-good indices. Consequentially, there are at least $\ell = \lceil \alpha n'/2 \rceil - j_0$ many j_0-super-good indices in every good row of Z, and hence exactly ℓ many (j_0, ℓ)-good indices.

We consider the last $b' = \lceil \alpha b/2 \rceil$ such "good rows" whose row numbers are denoted by $i_1, \ldots, i_{b'}$, where $b \geq i_1 > \cdots > i_{b'} \geq 1$. Obviously, for every $j \in [b']$, i_j is a function of (x, y), and more specifically, it is fully determined by x and $y_b, \ldots, y_{i_j+1}$ as mentioned in Remark 13.

For $i = 1, 2, \ldots, b$, we let $I_i = I_i(x, y)$ be the indicator that the Z-index (i, j_0) is (j_0, ℓ)-good in (x, y), i.e. $I_i(x, y) = 1$ if the X-index $(i, j_0 + y_i)$ is (j_0, ℓ)-good-in-(x, y) following Definition 12, and otherwise $I_i(x, y) = 0$. An important property of $I_i = I_i(x, y)$ is that when fixing x and $y_{i+1}, \ldots, y_b$, $I_i(x, y)$ becomes a univariate function of y_i. This is because the probability $p^{j_0}_{i,j_0+y_i}(x, y) = \Pr_{X,Y}\left[X_{i,j_0+y_i} = x_{i,j_0+y_i} \middle| \mathcal{E}_1(i-1, x),\ \mathcal{E}_2(i+1, j_0-1, x, y),\ \mathcal{E}_3(i, j_0+y_i-1, x_i)\right]$ is independent from $y_1, \ldots, y_{i-1}$.

For $i = i_1, i_2, \ldots, i_{b'}$, the indicator I_i equals to 1 with probability exactly ℓ/n' by Remark 13.

$$\Pr\left[I_i = 1 \middle| x, y_{i+1}, \ldots, y_b\right] = \Pr_{y_i \sim \mathcal{U}_{\log n'}}\left[(i, j_0 + y_i) \text{ is } (j_0, \ell)\text{-good in } (x, y) \middle| x, y_{i+1}, \ldots, y_b\right] = \ell/n'$$

Notice that $i_1 \geq i_2 + 1$ and I_{i_1} is already fully determined by $x, y_{i_1}, \ldots, y_b$. However, for every choice of $x, y_{i_2+1}, \ldots, y_b$ and uniformly chosen $y_{i_2} \sim \mathcal{U}_{\log n'}$, the indicator I_{i_2} is independent from I_{i_1} but has the same expectation as I_{i_1}. Similarly, we prove that the indicators $I_{i_1}, \ldots, I_{i_{b'}}$ are independent and identically distributed.

Then, we bound $\sum_{i=1}^{b} I_i$ with the following standard version of Chernoff bound.

Lemma 19 (Hoeffding's Inequality). *For independent almost surely bounded random variables $X_1, \ldots, X_n$, i.e. $\Pr[X_i \in [a_i, b_i]] = 1$ for $i \in [n]$, let $Y = X_1 + \cdots + X_n$. Then, for any positive Δ,*

$$\Pr\left[Y - \mathrm{E}[Y] \geq \Delta\right] \leq \exp\left(-\frac{2\Delta^2}{\sum_{i=1}^{n}(b_i - a_i)^2}\right)$$

□

With this lemma, the summation of I_i's are bounded as

$$\Pr_{y \sim \mathcal{U}_d}\left[\sum_{i=1}^{b} I_i \leq \alpha' b\right] \leq \Pr_{y \sim \mathcal{U}_d}\left[\sum_{k=1}^{b'} I_{i_k} \leq \alpha' b\right]$$
$$\leq \exp\Big(-2\big(\mathrm{E}\Big[\sum_{k=1}^{b'} I_{i_k}\Big] - \alpha' b\big)^2/b'\Big) = \exp\big(-2\big(b'\ell/n' - \alpha' b\big)^2/b'\big)$$

Plug in that $j_0 \leq \alpha n'/4$, $b' = \lceil \alpha b/2 \rceil$ and $\ell = \lceil \alpha n'/2 \rceil - j_0$ into the above inequality, and let the constant $\alpha' = \alpha^2/16$.

$$\Pr_{y \sim \mathcal{U}_d}\left[\sum_{i=1}^{b} I_i \leq \alpha' b\right] \leq \exp\big(-2\big(\alpha b/2 \cdot (\alpha n'/2 - j_0)/n' - \alpha' b\big)^2/(\alpha b/2)\big)$$
$$\leq \exp\Big(-\frac{4(\alpha^2/8 - \alpha')^2}{\alpha} \cdot b\Big) = \exp\big(-\alpha^3 b/64\big) = 2^{-\Omega(b)} \tag{5}$$

Thus, for the fixed x, $\Pr_{y \sim \mathcal{U}_d}\left[\sum_{i=1}^{b} I_i \geq \alpha' b\right] \geq 1 - 2^{-\Omega(b)} = 1 - \beta$.

In conclusion, for every fixed x that is α-informative for $j_0 \leq \alpha n'/4$, Z_{j_0} is (α', j_0, ℓ)-good within $\beta = \exp(-\alpha^3 b/64) = 2^{-\Omega(b)}$ for $\alpha' = \alpha^2/16 = \Omega(1)$ following Definition 16 of (α', j_0, ℓ)-good blocks on page 20.

□

2.5.5 Proof of Lemma 18 – from good blocks to next-block-min-entropy

Lemma 18 (restated). If there is a constant $\alpha' > 0$ such that the block Z_{j_0} is (α', j_0, ℓ)-good, then

$$\mathsf{H}_\infty\left[Z_{j_0} \middle| Z_1, \ldots, Z_{j_0-1}\right] \geq \alpha' b = \Omega(b)$$

If Z_j is (α', j, ℓ)-good for every $j = 1, 2, \ldots, j_0$, then $(Z_1, \ldots, Z_{j_0})$ has next-block-min-entropy $\Omega(b)$ in every block.

Proof. It suffices to prove the case of j_0. That is, for every fixed $z_{j_0} = (z_{1,j_0}, \ldots, z_{b,j_0}) \in \{0,1\}^b$, and for every $\mathsf{w} \in \{0,1\}^{b\times(j_0-1)}$,

$$\Pr\left[Z_{j_0} = z_{j_0} \middle| Z_1 \ldots Z_{j_0-1} = \mathsf{w}\right] \leq 2^{-\alpha' b}$$

For $i \in \{0, 1, \ldots, b\}$ and $t = 0, \ldots, i$, we denote by $a_{t,i}$ the conditional probability that for $(X, Y) \sim (\mathcal{D}, \mathcal{U}_d)$, the first i positions of Z_{j_0} agree with z_{j_0} (i.e. $Z_{1,j_0} = z_{1,j_0}, \ldots, Z_{i,j_0} = z_{i,j_0}$) and exactly t of those i many Z-indices are (j_0, ℓ)-good in (X, Y), where the condition is $Z_1 \ldots Z_{j_0-1} = \mathsf{w}$. Formally,

$$\begin{aligned} a_{t,i} = \Pr_{X\sim\mathcal{D}, Y\sim\mathcal{U}_d} \big[&\text{exactly } t \text{ of indices } (1, j_0), \ldots, (i, j_0) \text{ are } (j_0, \ell)\text{-good in } (X, Y) \\ &\wedge Z_{1,j_0} = z_{1,j_0} \wedge \ldots \wedge Z_{i,j_0} = z_{i,j_0} \big| Z_1 \ldots Z_{j_0-1} = \mathsf{w}\big] \end{aligned} \tag{6}$$

Claim 20. $\sum_{t=0}^{i} a_{t,i} \cdot 2^t \leq 1$ *for every i, $0 \leq i \leq b$.*

Proof of the claim. The claim is proved by induction on i. The base case $i = 0$ is trivial since $a_{0,0} = 1$. Assuming the case of i is known, it suffices to show $\sum_{t=0}^{i+1} a_{t,i+1} \cdot 2^t \leq \sum_{t=0}^{i} a_{t,i} \cdot 2^t$.

Recall the Definition 10 of y-order (and $\overline{y}_i$-order) on page 16. We consider the prefix r_i of $(z)_y$ under the $\overline{y}_{i+1}$-order of the following form.

$$r_i = \mathsf{w}_0 z_{1,j_0} \mathsf{w}_1 z_{2,j_0} \mathsf{w}_2 z_{3,j_0} \ldots \mathsf{w}_{i-1} z_{i,j_0} \mathsf{w}_i$$

Note that for every $\mathsf{w} \in \{0,1\}^{b(j_0-1)}$, the conditional event $Z_1 \ldots Z_{j_0-1} = \mathsf{w}$ in (6) is equivalent to that w is a prefix of $(Z)_y$ under any (y, j_0)-order. Therefore, it suffices to consider only r_i which has prefix w.

We say that an Z-index $(i+1, j_0)$ is j_0-good in $(r_i \circ z_{i+1,j_0}, \overline{y}_{i+1})$ if the Z-index $(i+1, j_0)$ is j_0-good in every (x, y) that is consistent with $r_i \circ z_{i+1,j_0}$ under $\overline{y}_{i+1}$-order. This is well defined since the corresponding X-index is $(i+1, j_0 + y_{i+1})$ whose j_0-goodness $p^{j_0}_{i+1, j_0+y_{i+1}}(x, y)$ is fully determined by $r_i \circ z_{i+1,j_0}$ and $\overline{y}_{i+1}$. Therefore, $(i+1, j_0)$ is j_0-good in $(r_i \circ z_{i+1,j_0}, \overline{y}_{i+1})$ if and only if

$$\Pr_{X,Y}\left[Z_{i+1,j_0} = z_{i+1,j_0} \middle| Z(X,Y) \text{ has prefix } r_i \text{ under } \overline{y}_{i+1}\text{-order and } \overline{Y}_{i+1} = \overline{y}_{i+1}\right] \leq 1/2 \tag{7}$$

In order to prove $\sum_{t=0}^{i+1} a_{t,i+1} \cdot 2^t \leq \sum_{t=0}^{i} a_{t,i} \cdot 2^t$, it suffices to show that for the fixed $z_{j_0} = z_{1,j_0} \ldots z_{b,j_0}$, for every y and for every r_i that is consistent with y and z_{j_0}, the contribution of $(r_i, \overline{y}_{i+1})$ to the summation $\sum_{t=0}^{i} a_{t,i} \cdot 2^t$ upper bounds that of $(r_i, \overline{y}_{i+1})$ to $\sum_{t=0}^{i+1} a_{t,i+1} \cdot 2^t$.

Notice that every (x, y) contributes to at most one $a_{t,i}$, for $0 \leq t \leq i$. More specifically, if $Z_{j_0} = Z(x,y)_{j_0} = z_{j_0}$ and $Z_1 \ldots Z_{j_0-1} = \mathsf{w}$, then (x, y) contributes to $a_{t,i}$ for t equal to the number of (j_0, ℓ)-good-in-(x, y) indices among the first i positions of Z_{j_0}, and the contribution is $\Pr\left[(X,Y) = (x,y) \middle| Z_1 \ldots Z_{j_0-1} = \mathsf{w}\right]$; otherwise when $Z(x,y)_{j_0} \neq z_{j_0}$ or $Z_1 \ldots Z_{j_0-1} \neq \mathsf{w}$, the contribution is zero.

Then, we say that $(r_i, \overline{y}_{i+1})$ contributes to $a_{t,i}$ if any (x, y) consistent with $(r_i, \overline{y}_{i+1})$ contributes to $a_{t,i}$. Every $(r_i, \overline{y}_{i+1})$ contributes to exactly one $a_{t,i}$, since t is uniquely determined by the given r_i and $\overline{y}_i$. The contribution of $(r_i, \overline{y}_{i+1})$ to $a_{t,i}$ is the sum of contribution of all the (x, y) consistent with it, which is

$$\begin{aligned} p(r_i, \overline{y}_{i+1}) &= \Pr_{X,Y}\left[Z(X,Y) \text{ has prefix } r_i \text{ under } \overline{y}_{i+1}\text{-order and } \overline{Y}_{i+1} = \overline{y}_{i+1} \middle| Z_1 \ldots Z_{j_0-1} = \mathsf{w}\right] \\ &= \Pr_{X,Y}\left[Z(X,Y) \text{ has prefix } r_i \text{ under } \overline{y}_{i+1}\text{-order and } \overline{Y}_{i+1} = \overline{y}_{i+1}\right] \end{aligned}$$

The condition $Z_1 \dots Z_{j_0-1} = \mathsf{w}$ can be omitted since it is implied from the event "$Z(X,Y)$ has prefix r_i" as long as w is a prefix of r_i.

On the other hand, we notice that for $0 \le t \le i+1$, the contribution of $(r_i, \overline{y}_{i+1})$ to $a_{t,i+1}$ is the same as $(r_i \circ z_{i+1,j_0}, \overline{y}_{i+1})$ to $a_{t,i+1}$, since (x,y) contributes positively only when $Z(x,y)_{i+1,j_0} = z_{i+1,j_0}$. Therefore, it suffices to consider the contribution of $(r_i \circ z_{i+1,j_0}, \overline{y}_{i+1})$ instead.

If $(i+1, j_0)$ is j_0-good in $(r_i \circ z_{i+1,j_0}, \overline{y}_{i+1})$, then recalling (7), we upper bound $p(r_i \circ z_{i+1,j_0}, \overline{y}_{i+1})$ by $p(r_i, \overline{y}_{i+1})/2$ as follows.

$$\begin{aligned}
& p(r_i \circ z_{i+1,j_0}, \overline{y}_{i+1}) \\
=& \Pr_{X,Y}\left[Z(X,Y) \text{ has prefix } r_i \circ z_{i+1,j_0} \text{ under } \overline{y}_{i+1}\text{-order and } \overline{Y}_{i+1} = \overline{y}_{i+1}\right] \\
=& \Pr_{X,Y}\left[Z_{i+1,j_0} = z_{i+1,j_0} \middle| Z(X,Y) \text{ has prefix } r_i \text{ under } \overline{y}_{i+1}\text{-order and } \overline{Y}_{i+1} = \overline{y}_{i+1}\right] \\
& \cdot \Pr_{X,Y}\left[Z(X,Y) \text{ has prefix } r_i \text{ under } \overline{y}_{i+1}\text{-order and } \overline{Y}_{i+1} = \overline{y}_{i+1}\right] \\
\le& \frac{1}{2} \Pr_{X,Y}\left[Z(X,Y) \text{ has prefix } r_i \text{ under } \overline{y}_{i+1}\text{-order and } \overline{Y}_{i+1} = \overline{y}_{i+1}\right] = p(r_i, \overline{y}_{i+1})/2
\end{aligned}$$

For any $(r_i, \overline{y}_{i+1})$ that contributes $p(r_i, \overline{y}_{i+1})$ to $a_{t,i}$, we discuss the following two cases:

- If $(i+1, j_0)$ is not j_0-good in $(r_i \circ z_{i+1,j_0}, \overline{y}_{i+1})$, then $(r_i \circ z_{i+1,j_0}, \overline{y}_{i+1})$ contributes to $a_{t,i+1}$ with at most $p(r_i, \overline{y}_{i+1})$.
- Otherwise, $(i+1, j_0)$ is j_0-good in $(r_i \circ z_{i+1,j_0}, \overline{y}_{i+1})$, then $(r_i \circ z_{i+1,j_0}, \overline{y}_{i+1})$ may contribute to either $a_{t,i+1}$ or $a_{t+1,i+1}$, depending on whether the Z-index $(i+1, j_0)$ is furthermore (j_0, ℓ)-good in (x,y) that is consistent with $(r_i \circ z_{i+1,j_0}, \overline{y}_{i+1})$. However, the total contribution of $(r_i \circ z_{i+1,j_0}, \overline{y}_{i+1})$ to $a_{t,i+1}$ and $a_{t+1,i+1}$ is at most $p(r_i \circ z_{i+1,j_0}, \overline{y}_{i+1}) \le p(r_i, \overline{y}_{i+1})/2$.

In conclusion, the contribution of $(r_i \circ z_{i+1,j_0}, \overline{y}_{i+1})$ to $\sum_{t=0}^{i+1} a_{t,i+1} \cdot 2^t$ is no more than $2^t p(r_i, \overline{y}_{i+1})$, which is the contribution of $(r_i, \overline{y}_{i+1})$ to $\sum_{t=0}^{i} a_{t,i} \cdot 2^t$. Therefore,

$$\sum_{t=0}^{i+1} a_{t,i+1} \cdot 2^t \le \sum_{t=0}^{i} a_{t,i} \cdot 2^t \le 1$$

Then, by induction we prove this claim. □

Let $i = b$ in the above claim, then $\sum_{t=0}^{b} a_{t,b} \cdot 2^t \le 1$, and in particular $\sum_{t=\lceil \alpha' b \rceil}^{b} a_{t,b} \le 2^{-\alpha' b}$. For every Z_{j_0} that is (α', j_0, ℓ)-good, we have $a_{0,b} = a_{1,b} = \cdots = a_{\lceil \alpha' b \rceil - 1, b} = 0$, since there are always at least $\alpha' b$ many (j_0, ℓ)-good indices in Z_{j_0} for every support of (X,Y). Thus, for every $z_{j_0} \in \{0,1\}^b$, and for every $\mathsf{w} \in \left(\{0,1\}^b\right)^{j_0-1}$,

$$\begin{aligned}
& \Pr_{X,Y}\left[Z_{j_0} = z_{j_0} \middle| Z_1 \dots Z_{j_0-1} = \mathsf{w}\right] \\
=& \sum_{t=0}^{b} a_{t,b} = \sum_{t=\lceil \alpha' b \rceil}^{b} a_{t,b} \\
\le& 2^{-\alpha' b}
\end{aligned}$$

Thus we prove that $\mathsf{H}_\infty[Z_{j_0} | Z_1 \dots Z_{j_0-1}] \ge \alpha' b = \Omega(b)$. □

2.6 Lower bounds for streaming extractors

We present the lower bound on the number of passes for all general (Section 2.6.2) and oblivious (Section 2.6.3) streaming extractors. Recall that we consider free access to the random seed, which makes the lower bound even stronger.

Theorem 21 (Lower bound for arbitrary extractors)**.** *Fix arbitrary constant $\lambda \geq 1$. Suppose* $\mathsf{Ext} : (\{0,1\}^n)^\lambda \times \{0,1\}^d \rightarrow \{0,1\}^m$ *is a ε-extractor for λ-many bit-fixing sources each of min-entropy k, and* Ext *is computable by a streaming algorithm with t streams and local memory size s, such that $t = \mathrm{O}(1)$, $n - k \geq \Omega(n)$, $k > m = s \cdot (\log \frac{1}{\varepsilon})^{\Omega(1)}$ and $d \leq \mathrm{O}(s)$. Then, the streaming algorithm must make at least $p = \Omega\left(\log\log \frac{1}{\varepsilon}\right)$ passes.*

Definition 22. *An* oblivious streaming extractor *is a streaming extractor that, after fixing the input length and the random seed, has its head move on the streams depending only the time step.*

In other words, there is a predetermined sequence according to which the head moves on streams.

Theorem 23 (Lower bound for oblivious extractors)**.** *Suppose* $\mathsf{Ext} : \{0,1\}^n \times \{0,1\}^r \rightarrow \{0,1\}^m$ *is a (k, ε)-extractor computable obliviously with $p = o(\log n)$ passes over $t = \mathrm{O}(1)$ streams and uses local memory of size s. For every $p = o(\log n), k = n^{1-\alpha}, m \geq k^{1-\beta}, r \leq \frac{m}{2t^{2p}} = m^{1-o(1)}$ and every constants $\alpha, \beta \in (0,1), \varepsilon < \frac{1}{2}$, we have that $s \geq m^{\Omega(1)} = n^{\Omega(1)}$. Moreover, this holds for bit-fixing sources.*

Remark. This lower bound indicates that constructing a streaming extractor for sources of min-entropy $o(n)$ with sub-logarithmic number of passes is far from what we know today. It is hard to imagine how an extractor can intelligently adapt its computation based on the specific content of the given sample.

These lower bounds rely on an analysis of the information flow in the streaming computation. Our analysis precisely captures the power of the streaming model. At a very high level, we first partition the stream into blocks and then quantify the information flow from input blocks to output blocks. Roughly speaking, if we only make very few passes, then there exists an output block that cannot collect a sufficient amount of entropy (even when using many streams) from an adversarially chosen input source. Thus, the output is statistically far from uniform.

In what follows, we introduce the framework to analyze the information flow. Then, we demonstrate our technique in the proofs of Theorem 21 and 23.

2.6.1 Dependency graphs and dependency trees

The concept of dependency graphs was originally introduced in [7] based on the treatment in [8, 49]. Our methodology uses these concepts in a quite different way than in previous work, i.e. we consider the growth of the entropy inside a single block rather than the sortedness across different blocks.

Let an (s, t, p) streaming algorithm have $p + 1$ phases induced by the p passes: whenever one pass ends on any of the streams the computation enters a new phase. The crucial observation in [49] is that when writing to a particular cell in the i-th phase, what is written only depends on the local memory together with the t cells currently being scanned by the t heads. Moreover, those t cells are written before the i-th phase, since no cell can be visited twice in a single phase.

Definition 24. *Let G be a deterministic streaming algorithm such that on input x, G makes at most p passes over t external tapes. The* dependency graph*, denoted by $\Gamma(x)$, is a directed $(p+1)$-layered graph associated with the computation of $G(x)$ as follows. Level i is associated with the i-th layer in $\Gamma(x)$ and it consists of all nodes labeled (v, i) if and only if tape cell v has ever been visited on or before the i-th phase*[4]*. $\Gamma(x)$ has an edge $(u, i) \rightarrow (v, i{+}1)$ if and only if any head is reading cell u when v is being written in the $(i{+}1)$-st phase. Furthermore, there is always an edge $(u, i) \rightarrow (u, i+1)$ as long as (u, i) is in $\Gamma(x)$ and $i \leq p$. The* dependency tree *rooted at v is the subgraph of all nodes in $\Gamma(x)$ with a directed path to $(v, p+1)$.*

[4]For completeness we assume that all input cells are visited before the first phase.

In Appendix 2.6.3 we give a concrete example illustrating the concepts of a dependency graph and a dependency tree.

Each layer in $\Gamma(x)$ corresponds to a single phase in the computation of $G(x)$ and every node has in-degree at most t. We also remark that not all old passes (over the remaining external streams) are necessarily finished when a new phase begins. In the new phase the algorithm will continue to process old passes.

Finally, we generalize the definition of dependency trees/graphs for blocks. The *blocks* are "super nodes" in $\Gamma(x)$ by merging nodes with the same dependency. The following partition is well-defined since the dependencies on every streaming tape change in some sense monotonically in each phase.

Definition 25. *A* block *is an equivalence class consisting of all nodes corresponding to tape cells at the same level on the same tape such that they depend on exactly the same set of blocks at the previous level. Specifically, an* input block *refers to a set of nodes at the first level corresponding to consecutive tape cells on the input tape.*

Henceforth, we abuse notation and let $\Gamma(x)$ be the generalized dependency graph whose nodes corresponds to blocks as in Definition 25. Intuitively, blocks are used to package the entropy from the input, and $\Gamma(x)$ describes the information flow during the computation except from the bits stored in the local memory. Later on, when proving the theorem, we will introduce a concrete way that formalizes the idea that dependencies of blocks in $\Gamma(x)$ correspond to statistical dependencies in the output.

Let the input x be partitioned into b input blocks $x = (x_1, x_2, \cdots, x_b)$. A corollary of Proposition 3.1 in [7] can be used to bound the number of blocks.

Lemma 26. (Corollary of Proposition 3.1 in [7]). *For any (s, t, p) streaming algorithm, if we partition its input x into b blocks and with $\Gamma(x)$ its dependency graph, then the number of blocks at level i in $\Gamma(x)$ is $\leq t^{i-1}b$.*

2.6.2 Proof of Theorem 21 – the general lower bound for randomness extractors

Recall the streaming computation for Ext, which makes in total p passes over $t = \mathrm{O}(1)$ streaming tapes and it uses local memory of size s. If $p = \Omega(\log n)$, then we have $p = \Omega\left(\log\log\frac{1}{\varepsilon}\right)$ even for $\varepsilon = 2^{-n^2}$. This is sufficient for every reasonable $\varepsilon > 0$ since the input length to Ext is $\mathrm{O}(n)$. From now on we only consider the case where $p = o(\log n)$. Also, recall that access to the random seed is given for free.

To construct a nemesis weak source we first introduce the parameters specifying the dependency graph $\Gamma(x^{(1)}, \ldots, x^{(\lambda)}, y)$ induced by the computation of $\mathsf{Ext}(x^{(1)}, \ldots, x^{(\lambda)}, y)$, where recall that the $x^{(i)}$'s come from the weak sources and y is the random seed. $\Gamma(x^{(1)}, \ldots, x^{(\lambda)}, y)$ is uniquely specified by $\mathsf{Ext}(x^{(1)}, \ldots, x^{(\lambda)}, y)$ since the computation of Ext is deterministic. Let $\ell = t^p = n^{o(1)}$ upper bound the number of leaf nodes in every dependency tree in $\Gamma(x^{(1)}, \ldots, x^{(\lambda)}, y)$, where each leaf node is an input block. We partition the length-λn input equally into $b = \left\lceil \frac{\lambda \ell n}{n-k-\log(1+\lambda)-t^p(p+1)(p\log t+\log n)} \right\rceil = \mathrm{O}(\lambda\ell) = o(n)$ blocks each of size $\lambda n/b$, where for simplicity from this point on we assume that $b|n$. Then, there are at most $b_O = t^p b$ output blocks by Lemma 26. Furthermore, the number g of different choices of dependency tree is upper bounded as follows.

$$g \leq \prod_{i=1}^{p+1} (bt^{i-1})^{t^{p+1-i}} < (b_O)^{l(p+1)} = (t^p b)^{t^p(p+1)}$$

Now, we define a function $F : \{0,1\}^{\lambda n} \times \{0,1\}^d \to [g]$ such that for every $x^{(1)}, \ldots, x^{(\lambda)} \in \{0,1\}^n, y \in \{0,1\}^d$, $F(x^{(1)}, \ldots, x^{(\lambda)}, y) = z$, where z is the distinct index of the dependency tree rooted at the largest[5] output block A in the dependency graph $\Gamma(x^{(1)}, \ldots, x^{(\lambda)}, y)$. Such a function F is well-defined since $\Gamma(x^{(1)}, \ldots, x^{(\lambda)}, y)$ is unique for fixed $(x^{(1)}, \ldots, x^{(\lambda)}, y)$. Moreover, since A is the largest among the b_O many output blocks, it contains at least m/b_O output bits.

[5]If there are more than one block being largest then we can choose i as the index of any of them.

By averaging there are at least $\frac{2^{\lambda n+d}}{g}$ tuples $(x^{(1)},\ldots,x^{(\lambda)},y)$ sharing the same output z_0 under F. Then, by a simple probabilistic argument we conclude that there exist sets $S'_1,\cdots,S'_\lambda \subseteq \{0,1\}^n$, $|S'_i| \geq \frac{2^n}{(1+\lambda)g}$ such that for every $x = \left(x^{(1)},\ldots,x^{(\lambda)}\right) \in S' = S'_1 \times \cdots \times S'_\lambda$ there exists $R_x \subseteq \{0,1\}^d$ satisfying $|R_x| \geq \frac{2^d}{(1+\lambda)g}$ and for all $y \in R_x, F(x^{(1)},\ldots,x^{(\lambda)},y) = z_0$. For convenience, we denote by $\mathsf{Tree}(z_0)$ the dependency tree indexed by z_0.

Now we lower bound the number of $x \in S'$ inducing identical content in all the leaf nodes $\mathsf{Tree}(z_0)$. Recall that every dependency tree has at most ℓ leaf nodes (input blocks), in particular at most $\ell \cdot \frac{\lambda n}{b}$ input bits are contained in the leaf nodes of $\mathsf{Tree}(z_0)$. Moreover, their indices are fully determined by z_0. Let D_{z_0} be the set consisting of those indices, then $|D_{z_0}| \leq \frac{\lambda n \ell}{b}$ and $x|_{D_{z_0}}$ contains exactly the content of all leaf nodes of $\mathsf{Tree}(z_0)$ on input x. Partition $D_{z_0} = \left(D^{(1)}_{z_0},\ldots,D^{(\lambda)}_{z_0}\right)$ such that $D^{(i)}_{z_0}$ is the part of D_{z_0} consistent with $x^{(i)}$.

For every i by averaging there is $x^{(i)}_0 \in \{0,1\}^{|D^{(i)}_{z_0}|}$ and a sufficiently large subset $S''_i \subseteq S'_i$, such that $|S''_i| \geq \frac{|S'_i|}{2^{|D^{(i)}_{z_0}|}}$ and moreover if $x \in S'' = S''_1 \times \cdots \times S''_\lambda$ then the values of the bits in leaf nodes of $\mathsf{Tree}(z_0)$ is fixed to $x_0 = \left(x^{(1)}_0,\ldots,x^{(\lambda)}_0\right)$. Formally, $|S''| \geq \prod_{i=1}^{\lambda} \frac{|S'_i|}{2^{|D^{(i)}_{z_0}|}} \geq \frac{|S'|}{2^{|D_{z_0}|}} \geq \frac{|S'|}{2^{\lambda \ell n/b}}$ and $x|_{D_{z_0}} = x_0$ for every $x \in S''$.

Recalling that for every $x \in S'$ there exists $R_x \subseteq \{0,1\}^d, |R_x| \geq \frac{2^d}{(1+\lambda)g}$, such that for every $y \in R_x$, the dependency tree rooted at the largest output block A is $\mathsf{Tree}(z_0)$. If furthermore $x \in S''$, then $\mathsf{Tree}(z_0)$ has only $x|_{D_{z_0}} = x_0$ in its leaf nodes. That is, for every $x \in S''$ and every $y \in R_x$, the largest output block A acquires all its information entropy from the local memory during the computation, since the input blocks it depends on have fixed content. Note that there are no more than t^p intermediate nodes in $\mathsf{Tree}(z_0)$, while to each node at most s bits entropy can be transferred from local memory. Thus, for such x and y, A acquires at most $t^p s$ bits information entropy during the entire computation.

Recalling that $b = \left\lceil \frac{\lambda \ell n}{n-k-\log(1+\lambda)-t^p(p+1)(p\log t+\log n)} \right\rceil \geq \frac{\lambda \ell n}{n-k-\log(1+\lambda)-\log g}$, we have $|S''_i| \geq \frac{|S'_i|}{2^{\lambda \ell n/b}} \geq \frac{2^n}{(1+\lambda)g \cdot 2^{\lambda \ell n/b}} \geq 2^k$.

Now, we are ready to present the nemesis bit-fixing sources. We denote by $\mathcal{U}_{S_i}$ the uniform distribution over $S_i = \left\{x^{(i)} \in \{0,1\}^n \,\middle|\, x^{(i)}|_{D^{(i)}_{z_0}} = x^{(i)}_0\right\}$. Note that $\mathcal{U}_{S_i}$ is bit-fixing by definition, and since $2^k \leq |S''_i| \leq |S_i| = 2^{n-|D^{(i)}_{z_0}|}$, $\mathcal{U}_{S_i}$ is also an (n,k)-source. Let $S = S_1 \times \cdots \times S_\lambda$, then $\mathcal{U}_S$ is the corresponding product distribution.

Finally, we lower bound the statistical distance between $\mathsf{Ext}(\mathcal{U}_S,\mathcal{U}_d)$ and $\mathcal{U}_m$.

Fix arbitrary $z_0 \in [g]$. Denote by $\mathcal{E} = \mathcal{E}(x,y)$ the event $F(x,y) = z_0$. Since for every $x \in S'' \subseteq S$ we have $R_x \subseteq \{0,1\}^d$ such that $|R_x| \geq \frac{2^d}{(1+\lambda)g}$ and for every $y \in R_x$ there is $F(x,y) = z_0$, and recalling that $|S'_i| \geq \frac{2^n}{(1+\lambda)g}$,

$$\begin{aligned}
\Pr_{x\leftarrow\mathcal{U}_S,y\leftarrow\mathcal{U}_d}[\mathcal{E}(x,y)] &\geq \Pr_{x\leftarrow\mathcal{U}_S}[x \in S''] \cdot \Pr_{y\leftarrow\mathcal{U}_d}[\mathcal{E}(x,y) \mid x \in S''] \\
&\geq \frac{|S''|}{|S|} \cdot \min_{x\in S''} \Pr_{y\leftarrow\mathcal{U}_d}[y \in R_x] \\
&\geq \frac{|S'|/2^{|D_{z_0}|}}{2^{\lambda n-|D_{z_0}|}} \cdot \min_{x\in S''} \frac{|R_x|}{2^d} \geq \frac{|S'|}{2^{\lambda n}} \cdot \frac{1}{(1+\lambda)g} = \frac{1}{(1+\lambda)^{1+\lambda} g^{1+\lambda}}
\end{aligned}$$

On the other hand, the possible outputs of A conditioned on $\mathcal{E}$ are at most $2^{t^p s}$, since A is fully determined by the $\leq t^p s$ bits from the local memory. By conditioning on $\mathcal{E}$ and taking into account the position of A in the output there are $2^{t^p s} m$ distinct choices for A while there are at most $2^{m-m/b_O}$ possibilities for the rest $m - m/b_O$ bits in the output. Thus, there are at most $2^{t^p s} m \cdot 2^{m-m/b_O}$ possible outputs conditioned on $\mathcal{E}$ (by union bound). Let $Z \subseteq \{0,1\}^m$ be defined as

$$Z = \left\{z \in \{0,1\}^m \mid \exists x \in \{0,1\}^{\lambda n}, \exists y \in \{0,1\}^d, \text{such that } F(x,y) = z_0, \mathsf{Ext}(x,y) = z\right\}$$

It immediately follows that $|Z| \leq 2^{t^p s} m \cdot 2^{m-m/b_O}$, and in fact $Z = \{\mathsf{Ext}(x,y) \mid \mathcal{E}(x,y)\}$ is the range of Ext given that $\mathcal{E}(x,y)$ holds.

Therefore, the statistical distance between $\mathsf{Ext}(\mathcal{U}_S,\mathcal{U}_d)$ and $\mathcal{U}_m$ is lower bounded by

$$\begin{aligned}
\mathcal{SD}\left(\mathsf{Ext}(\mathcal{U}_S,\mathcal{U}_d),\mathcal{U}_m)\right) &= \frac{1}{2}\sum_{z\in\{0,1\}^m} |\Pr[\mathsf{Ext}(\mathcal{U}_S,\mathcal{U}_d) = z] - \Pr[\mathcal{U}_m = z]| \\
&\geq \frac{1}{2}\sum_{z\in Z}\left(\Pr[\mathsf{Ext}(\mathcal{U}_S,\mathcal{U}_d) = z] - \frac{1}{2^m}\right) = \frac{1}{2}\left(\Pr[\mathsf{Ext}(\mathcal{U}_S,\mathcal{U}_d) \in Z] - \frac{|Z|}{2^m}\right) \\
&\geq \frac{1}{2}\left(\Pr_{x\leftarrow\mathcal{X},y\leftarrow\mathcal{U}_d}[\mathcal{E}(x,y)] - \frac{|Z|}{2^m}\right) \\
&\geq \frac{1}{2(1+\lambda)^{1+\lambda}g^{1+\lambda}} - \frac{2^{t^p s} m \cdot 2^{m-m/b_O}}{2^{m+1}} \\
&\geq \frac{1}{2(1+\lambda)^{1+\lambda}(t^p b)^{t^p(p+1)(1+\lambda)}} - 2^{t^p s + \log m - 1 - m/b_O}
\end{aligned}$$

Since Ext is a (k,ε)-extractor, $\varepsilon \geq \mathcal{SD}\left(\mathsf{Ext}(\mathcal{U}_S,\mathcal{U}_d),\mathcal{U}_m)\right)$ by definition. Thus, when $m/b_O = \omega(t^p s + t^p(p+1)(p\log t + \log b))$

$$\varepsilon \geq \Omega\left(\frac{1}{(t^p b)^{2t^p(p+1)(1+\lambda)}}\right)$$

In conclusion, we get $p = \Omega\left(\log\log(\frac{1}{\varepsilon})\right)$ for every $m = s \cdot (\log\frac{1}{\varepsilon})^{\Omega(1)}$. □

2.6.3 Proof of Theorem 23 – the limitation of oblivious extractors

We give a lower bound for oblivious streaming extractors, which also holds for bit-fixing sources. This is a logarithmic lower bound on the number of passes for local memory size $s = n^{o(1)}$. This shows an exponential separation between non-oblivious and the oblivious RRB extractor that makes $\log\log(1/\varepsilon)$-passes.

Given Ext, we construct an adversarial bit-fixing source to show the lower bound. We fix the longest output block, and make all the entropy in the bit-fixing source concentrate on the bits that are least frequently associated to the specific output block (over the randomness of the seed). Therefore, this output block cannot acquire sufficient information from the input, which induces significant distance from the uniform distribution.

The following theorem implies Theorem 23.

Theorem 27. *Suppose* $\mathsf{Ext} : \{0,1\}^n \times \{0,1\}^r \to \{0,1\}^m$ *is a* (k,ε)*-extractor for bit-fixing sources and it is computable with* p *passes over* t *streams and using local memory* s*. If* Ext *is randomized oblivious, i.e. for fixed random seed* y, $\mathsf{Ext}_y(\cdot) = \mathsf{Ext}(\cdot,y)$ *is computable by an oblivious streaming algorithm, then for every* $k = o\left(\frac{n}{t^p}\right), p = o(\log n)$ *and constant* $\varepsilon < \frac{1}{2}$, $t = \mathrm{O}(1)$*, there must be* $s > \frac{m - t^p br - t^p b}{t^{2p} b}$ *for* $b > \frac{t^p}{1-2\varepsilon-kt^p/n} = \mathrm{O}(t^p)$.

Proof. First we consider the dependency graph $\Gamma(x,y)$ with the input equally partitioned into b blocks, where each block has length n/b. By Lemma 26, there are at most $t^p b$ output blocks and hence the longest output block, denoted by A, contains at least $\frac{m}{t^p b}$ bits. Then, there are $c = t^p$ leaf nodes in the dependency tree T_A rooted at A, since it is a t-branching tree with depth p.

Going over all 2^r choices of random seeds, every specific input block would be hit $\frac{2^r c}{b}$ times in average one input block appears as a leaf node in T_A for $\leq \frac{2^r c}{b}$ distinct random seeds (since a single seed may induce a T_A hitting the same input block multiple times). Thus, there exists $\left\lceil\frac{kb}{n}\right\rceil$ input blocks that are hit by at most $\left(\left\lceil\frac{kb}{n}\right\rceil\frac{c}{b}\right)\cdot 2^r$ distinct random seeds.

Suppose the input is a bit-fixing source X with all its entropy concentrated in those $\left\lceil\frac{kb}{n}\right\rceil$ input blocks. Then, only $\leq \left(\left\lceil\frac{kb}{n}\right\rceil\frac{c}{b}\right)\cdot 2^r$ distinct random seeds are able to induce T_A with at least one unfixed leaf node. That is,

with probability $1 - \left\lceil \frac{kb}{n} \right\rceil \frac{c}{b}$, all the leaf nodes in T_A are fixed. In such case, A is fully determined by the local memory when the heads enter any intermediate nodes in T_A, which is bounded by $t^p s$ since there are less than t^p intermediate nodes in T_A. Thus, A is fully determined by the $t^p s$ bits from local memory and r bits from the seed. For this $1 - \left\lceil \frac{kb}{n} \right\rceil \frac{c}{b}$ fraction of random seeds, each possible output in A appears with probability at least $\frac{1}{2^{r+t^p s}}$.

Finally, we lower bound the statistical distance between $\mathsf{Ext}(X, \mathcal{U}_r)$ and uniform distribution with their restriction on the output block A,

$$
\begin{aligned}
&\mathcal{SD}\left(\mathsf{Ext}(X,\mathcal{U}_r),\mathcal{U}_m\right)\\
\geq&\mathcal{SD}\left(\mathsf{Ext}(X,\mathcal{U}_r)|_A,\mathcal{U}_{|A|}\right)\\
\geq&\left(1-\left\lceil\frac{kb}{n}\right\rceil\frac{c}{b}\right)\cdot 2^r\cdot 2^{t^p s}\left(\frac{1}{2^{r+t^p s}}-\frac{1}{2^{|A|}}\right)\\
\geq&\left(1-\left\lceil\frac{kb}{n}\right\rceil\frac{c}{b}\right)\cdot 2^r\cdot 2^{t^p s}\left(\frac{1}{2^{r+t^p s}}-\frac{1}{2^{\frac{m}{t^p b}}}\right)\\
\geq&\left(1-\left\lceil\frac{kb}{n}\right\rceil\frac{c}{b}\right)\left(1-\frac{1}{2^{\frac{m}{t^p b}-r-t^p s}}\right)
\end{aligned}
$$

Assume for contradiction that $s \leq \frac{m - t^p br - t^p b}{t^{2p} b}$. Then, $\frac{m}{t^p b} - r - t^p s \geq 1$ and hence $\frac{1}{2^{\frac{m}{t^p b}-r-t^p s}} \leq \frac{1}{2}$. Plugging it into the lower bound of $\mathcal{SD}\left(\mathsf{Ext}(X,\mathcal{U}_r),\mathcal{U}_m\right)$,

$$
\mathcal{SD}\left(\mathsf{Ext}(X,\mathcal{U}_r),\mathcal{U}_m\right) \geq \frac{1}{2}\left(1-\left\lceil\frac{kb}{n}\right\rceil\frac{c}{b}\right) \geq \frac{1}{2} - \frac{kt^p}{2n} - \frac{t^p}{2b}
$$

which means $\mathcal{SD}\left(\mathsf{Ext}(X,\mathcal{U}_r),\mathcal{U}_m\right) > \varepsilon$ for every constant $\varepsilon < \frac{1}{2}$, and $k = o\left(\frac{n}{t^p}\right), p = o(\log n)$, and $b > \frac{t^p}{1-2\varepsilon-kt^p/n} = \mathrm{O}\left(t^p\right)$. In conclusion, it must be the case $s > \frac{m-t^p br - t^p b}{t^{2p} b}$. $\square$

References

1. J. Katz and Y. Lindell. *Introduction to Modern Cryptography: Principles and Protocols*. Chapman & Hall/CRC, 2007.

2. M. P. Allen and D. J. Tildesley. *Computer Simulation of Liquids*. Oxford Science Publications, 1989.

3. K. Binder and D. Heermann. *Monte Carlo Simulation in Statistical Physics: An Introduction*. Springer, 2010.

4. C. Andrieu, N. De Freitas, A. Doucet, and M. I. Jordan. An introduction to MCMC for machine learning. *Machine learning*, 50(1-2):5–43, 2003.

5. R. Motwani and P. Raghavan. *Randomized Algorithms*. Cambridge University Press, 1995.

6. P. A. Papakonstantinou and G. Yang. Cryptography with streaming algorithms. In *CRYPTO (2)*, pages 55–70, 2014.

7. P. Beame and T. Huynh. The value of multiple read/write streams for approximating frequency moments. *ACM Transactions on Computation Theory*, 3(2):6, 2012.

8. M. Grohe, A. Hernich, and N. Schweikardt. Lower bounds for processing data with few random accesses to external memory. *Journal of the ACM*, 56(3):Art. 12, 58, 2009.

9. Z. Bar-Yossef, O. Reingold, R. Shaltiel, and L. Trevisan. Streaming computation of combinatorial objects. In *Conference on Computational Complexity (CCC)*, pages 133–142. IEEE, 2002.

10. S. Pironio, A. Acín, S. Massar, A B de La Giroday, Dzimitry N Matsukevich, P Maunz, S Olmschenk, D Hayes, L Luo, T A Manning, et al. Random numbers certified by Bell's theorem. *Nature*, 464(7291):1021–1024, 2010.

11. S. Pironio and S. Massar. Security of practical private randomness generation. *Phys. Rev. A*, 87:012336, 2013.

12. U. V. Vazirani and T. Vidick. Certifiable quantum dice - or, testable exponential randomness expansion. *Philosophical Transactions-Royal Society of London Series A*, 370:3432–3448, 2012.

13. M. Um, X. Zhang, J. Zhang, Y. Wang, S. Yangchao, D-L Deng, L.-M. Duan, and K. Kim. Experimental certification of random numbers via quantum contextuality. *Scientific reports*, 3, 2013.

14. X. Ma, F. Xu, H. Xu, X. Tan, B. Qi, and H.-K. Lo. Postprocessing for quantum random-number generators: Entropy evaluation and randomness extraction. *Phys. Rev. A*, 87(6):062327, 2013.

15. D. G. Marangon, G. Vallone, and P. Villoresi. Random bits, true and unbiased, from atmospheric turbulence. *Scientific Reports*, 2014.

16. A. Rukhin, J. Soto, J. Nechvatal, M. Smid, and E. Barker. A statistical test suite for random and pseudorandom number generators for cryptographic applications. Special publication 800–22 (revision 1a), National Institute of Standards and Technology, 2010.

17. The marsaglia random number CDROM including the Diehard Battery of Tests of Randomness. 2008.

18. M. Bansal. Big data: Creating the power to move heaven and earth. *MIT Technology Review*, 2014.

19. J. Wakefield and P. Kerley. How "big data" is changing lives. *BBC (News Technology)*, 2013.

20. I. J Cox, M. L Miller, J. A Bloom, and C. Honsinger. *Digital watermarking*, volume 53. Springer, 2002.

21. R. Shaltiel. *Current trends in theoretical computer science. The Challenge of the New Century. (book chapter)*, volume Vol 1: Algorithms and Complexity. World Scientific, 2004.

22. L. Trevisan. Construction of extractors using pseudo-random generators. In *Symposium on Theory Of Computing (STOC)*, pages 141–148. ACM, ACM, 1999.

23. R. Raz, O. Reingold, and S. Vadhan. Extracting all the randomness and reducing the error in trevisan's extractors. In *Symposium on Theory Of Computing (STOC)*, pages 149–158. ACM, ACM, 1999.

24. G. Valiant and P. Valiant. Estimating the unseen: An n/log(n)-sample estimator for entropy and support size, shown optimal via new clts. In *Symposium on Theory Of Computing (STOC)*, pages 685–694. ACM, 2011.

25. J. von Neumann. Various techniques in connection with random digits. *Applied Math Series*, 12:36–38, 1951.

26. D. Zuckerman. Simulating bpp using a general weak random source. *Algorithmica*, 16(4-5):367–391, 1996.

27. B. Chor and O. Goldreich. Unbiased bits from sources of weak randomness and probabilistic communication complexity. *SIAM Journal on Computing*, 17(2):230–261, 1988.

28. K-M Chung, M. Mitzenmacher, and S. Vadhan. Why simple hash functions work: Exploiting the entropy in a data stream. *Theory of Computing*, 9(30):897–945, 2013.

29. J. Soto and L. Bassham. Randomness testing of the advanced encryption standard finalist candidates. Technical report, NIST (NISTIR) 6483, 2000.

30. A. Hájek. Interpretations of probability. In Edward N. Zalta, editor, *The Stanford Encyclopedia of Philosophy*. Winter 2012 edition, 2012.

31. B. Barak, R. Impagliazzo, and A. Wigderson. Extracting randomness using few independent sources. *SIAM Journal on Computing*, 36(4):1095–1118, 2006.

32. D. Zuckerman. General weak random sources. In *Foundations of Computer Science (FOCS)*, pages 534–543. IEEE, IEEE, 1990.

33. J. Ziv and A. Lempel. A universal algorithm for sequential data compression. *IEEE Transactions on information theory*, 23(3):337–343, 1977.

34. N. Nisan and D. Zuckerman. Randomness is linear in space. *Journal of Computer and System Sciences*, 52(1):43–52, 1996.

35. The templated portable i/o environment. 2013.

36. The gnu multiple precision arithmetic library. 2014.

37. Fast galois field arithmetic library in c/c++. 2007.

38. Mathematica. 2015.

39. M. Santha and U. V. Vazirani. Generating quasi-random sequences from semi-random sources. *Journal of Computer and System Sciences*, 33(1):75–87, 1986.

40. R. Shaltiel. How to get more mileage from randomness extractors. *Random Structures & Algorithms*, 33(2):157–186, 2008.

41. R. Raz. Extractors with weak random seeds. In *Symposium on Theory Of Computing (STOC)*, pages 11–20. ACM, 2005.

42. Y. Dodis, A. Elbaz, R. Oliveira, and R. Raz. Improved randomness extraction from two independent sources. In *RANDOM*, pages 334–344. Springer, 2004.

43. J. Bourgain. On the construction of affine extractors. *GAFA Geometric And Functional Analysis*, 17(1):33–57, 2007.

44. A. Gabizon, R. Raz, and R. Shaltiel. Deterministic extractors for bit-fixing sources by obtaining an independent seed. *SIAM Journal on Computing*, 36(4):1072–1094, 2006.

45. J. Kamp and D. Zuckerman. Deterministic extractors for bit-fixing sources and exposure-resilient cryptography. *SIAM Journal on Computing*, 36(5):1231–1247, 2006.

46. A. Rao. Extractors for low-weight affine sources. In *Conference on Computational Complexity (CCC)*, pages 95–101. IEEE, 2009.

47. I. Haitner, O. Reingold, and S. Vadhan. Efficiency improvements in constructing pseudorandom generators from one-way functions. In *Symposium on Theory Of Computing (STOC)*, pages 437–446, 2010.

48. S. P. Vadhan and C. J. Zheng. Characterizing pseudoentropy and simplifying pseudorandom generator constructions. In *Symposium on Theory Of Computing (STOC)*, pages 817–836, 2012.

49. M. Grohe and N. Schweikardt. Lower bounds for sorting with few random accesses to external memory. In *Symposium on Principles of Database Systems (PODS)*, pages 238–249, 2005.

50. J. Chen and C.-K. Yap. Reversal complexity. *SIAM Journal on Computing*, 20(4):622–638, 1991.

51. A. Hernich and N. Schweikardt. Reversal complexity revisited. *Theoretical Computer Science*, 401(1-3):191–205, 2008.

52. Z. Bar-Yossef, O. Goldreich, and A. Wigderson. Deterministic amplification of space-bounded probabilistic algorithms. In *Conference on Computational Complexity (CCC)*, pages 188–198. IEEE, 1999.

53. T. Hartman and R. Raz. On the distribution of the number of roots of polynomials and explicit weak designs. *Random Structures & Algorithms*, 23(3):235–263, 2003.

54. R. Shaltiel and C. Umans. Simple extractors for all min-entropies and a new pseudo-random generator. In *Foundations of Computer Science (FOCS)*, pages 648–657. IEEE, 2001.

55. R. Raz, O. Reingold, and S. Vadhan. Extracting all the randomness and reducing the error in trevisan's extractors. In *Symposium on Theory Of Computing (STOC)*, pages 149–158. ACM, ACM, 1999.

56. S. Arora and B. Barak. *Computational complexity: a modern approach*, volume 1. Cambridge University Press Cambridge, 2009.

57. S. P. Vadhan. Pseudorandomness. *Foundations and Trends in Theoretical Computer Science*, 7(1-3):1–336, 2012.

58. R. Impagliazzo, L. A. Levin, and M. Luby. Pseudo-random generation from one-way functions. In *Symposium on Theory Of Computing (STOC)*, pages 12–24. ACM, 1989.

59. R. Impagliazzo and D. Zuckerman. How to recycle random bits. In *Foundations of Computer Science (FOCS)*, pages 248–253. IEEE, 1989.

Appendix

A Streaming computable encoding of 2-universal hash functions

A family of 2-universal hash functions can be used as a randomness extractor by Lemma 6.

We denote by S_n^m a family of 2-universal hash functions from $\{0,1\}^n$ to $\{0,1\}^m$, and let H_n^m be the uniform distribution over S_n^m.

Recall that the family of random linear functions, i.e. $h(\mathbf{x}) = \mathbf{M}\mathbf{x}$ for $\mathbf{M} \in_R \mathbb{Z}_2^{n\times n}$, forms a family of 2-universal hash functions. Moreover, those random linear functions have a streaming computable randomized encoding [6], which is in particular a family of streaming computable 2-universal hash functions.

Claim 28. *Let S_n^m be a family of linear hash functions, i.e. every function $h : \{0,1\}^n \to \{0,1\}^m$ has the form $h(\mathbf{y}) = \mathbf{y}^T\mathbf{H}$, where $\mathbf{H} \in_R \{0,1\}^{n\times m}$ is specified by h. Then, every $h \in S_n^m$ has a streaming computable randomized encoding $\hat{h} : \{0,1\}^n \times \{0,1\}^{(n-1)m} \to \{0,1\}^{nm}$.*

Moreover, $S_{(n-1)m+n}^{nm} = \widehat{S_n^m} = \left\{\hat{h} | h \in S_n^m\right\}$ defines a family of streaming computable 2-universal hash functions from $(n-1)m+n$ bits to nm bits.

Proof. Suppose $\mathbf{y} = (y_1, \cdots, y_n)$, and $\mathbf{H}$ be as follows:

$$\mathbf{H} = \begin{bmatrix} h_{1,1} & h_{1,2} & \cdots & h_{1,m} \\ \vdots & \vdots & \vdots & \vdots \\ h_{n,1} & h_{n,2} & \cdots & h_{n,m} \end{bmatrix} \tag{8}$$

As a result, we have $\mathbf{y}^T\mathbf{H} = (\sum_{i=1}^n y_i h_{i,1}, \sum_{i=1}^n y_i h_{i,2}, \cdots, \sum_{i=1}^n y_i h_{i,m})$, where every output bit depends on all n bits in y. Since summation over GF[2] is equivalent to parity operation, hence in order to reduce the locality of the product $\mathbf{y}^T\mathbf{H}$, we can introduce a sequence of random bits $r \in \{0,1\}^{n\times m}$ to partition every term $\sum_{i=1}^n y_i h_{i,j}$ into smaller "masked pieces", for each $j = 1, 2, \cdots, m$.

Thus, we design $\hat{h} : \{0,1\}^n \times \{0,1\}^{(n-1)\times m} \to \{0,1\}^{n\times m}$ as

$$\begin{aligned} \hat{h}(\mathbf{y},\mathbf{r}) = \langle y_1 h_{1,1} + r_{1,1}, \quad & \cdots, \quad & y_1 h_{1,m} + r_{1,m}, \\ y_2 h_{2,1} + r_{1,1} + r_{2,1}, \quad & \cdots, \quad & y_2 h_{2,m} + r_{1,m} + r_{2,m}, \\ & \cdots & \\ y_n h_{n,1} + r_{n-1,1}, \quad & \cdots, \quad & y_n h_{n,m} + r_{n-1,m}\rangle. \end{aligned} \tag{9}$$

It is easy to verify that $\hat{h}$ is a perfect randomized encoding of the function $h(\mathbf{y}) = \mathbf{y}^T\mathbf{H}$, i.e. $\hat{h}(\mathbf{y},\mathbf{r})$ uniformly distributes over all possible encodings of $h(\mathbf{y})$.

It remains to explain how $\hat{h}$ can be computed by a streaming algorithm with constant many passes and external streams. By scanning $\mathbf{y}$ and $\mathbf{H}$ in a row-first order, we can fill in all terms like $y_i h_{i,j}$. With another two passes over $\mathbf{r}$ (and scanning $y_i h_{i,j}$'s simultaneously) we complete (9). □

B Example illustrating the dependency tree concept

We provide a concrete dependency graph example depicted on Figure S3. This is an example of a simple streaming algorithm that adds up two integers in binary. In this example the input is 11100 and 110. The associated dependency tree is depicted in Figure S4.

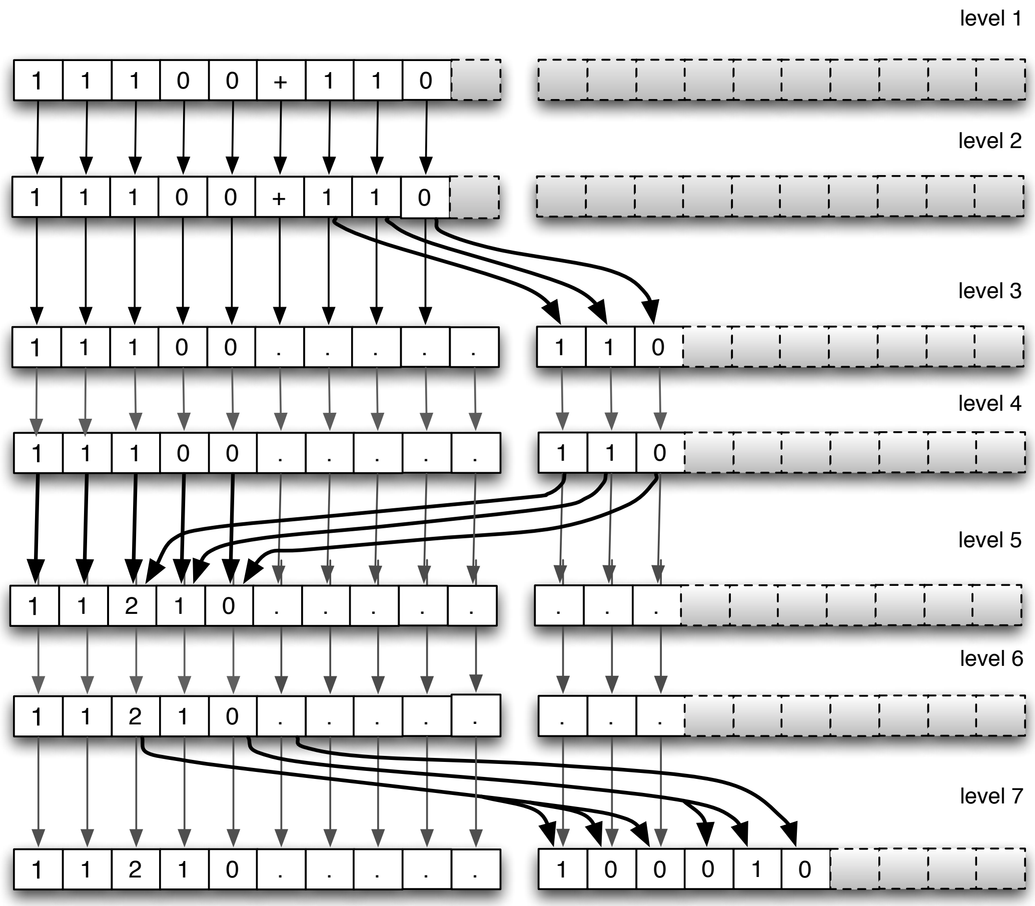

Figure S3: Example of a dependency graph for a natural streaming addition algorithm with two external tapes and seven levels. Each level in this graph consists of exactly the nodes corresponding to all tape cells visited before the associated pass begins.

In the dependency graph, level 1 represents the input "11100" and "110"; level 3 corresponds to copying the second input "110" to the second tape; level 5 adds up the two numbers "11100" and "110" without carries; level 7 deals with carries. Level 2, 4, and 6 are depicted only for completeness, since every time we begin two passes simultaneously, and as a result the actual passes associated with level 2, 4, and 6 are handled in level 3, 5, and 7 respectively.

The first six levels need no explanation. In the last level the algorithm maintains a counter in its local memory to count how many cells have been scanned before finding the next "0" or "2", and it writes $100 \cdots 0$ if reads "2" or $011 \cdots 1$ if reads "0", where the length of $100 \cdots 0$ (resp. $011 \cdots 1$) equals to the value stored in the counter. $\square$

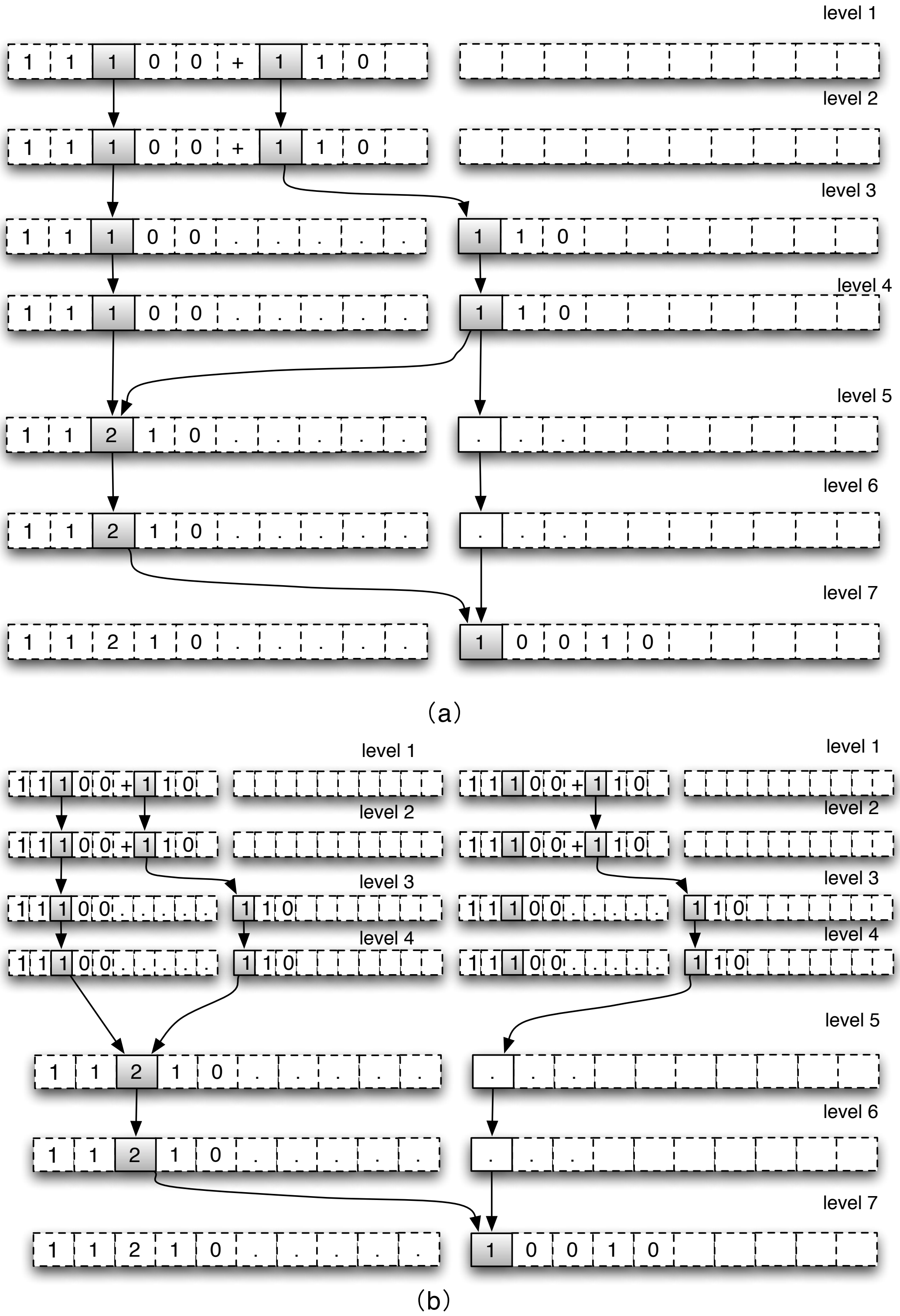

Figure S4: Example of a dependency tree assuming that the root v is the first output cell in Figure S3. Here, (a) is the *skeleton* of the dependency tree rooted at v, and (b) is the actual dependency tree of v. Note that (b) duplicates at level 4 to eliminate the shared children and transform the skeleton to a tree. In particular, Lemma 26 holds for the skeleton as well as for the corresponding dependency tree.

C The alternative streaming extractor for bit-fixing sources

We present another streaming extractor for bit-fixing sources, the random cyclic shift RCS extractor.

RCS first generates ab many copies of the input for $a = \mathrm{O}\left(\log \frac{1}{\varepsilon}\right)$ and $b = \mathrm{O}\left(\log n\right)$. Then, it performs random cyclic shifting independently on each of the ab copies, so that the entropy is equalized for every position in every copy. In the next step, RCS adds up the corresponding bits in every a shifted copies to get size-b blocks. Finally, RCS invokes a standard extractor, which can be realized as a random 2-universal hash function, on every size-b block.

Algorithm 29. *Let σ_1, σ_2 denote the two streams. The extractor $\mathsf{Ext} : \{0,1\}^n \times \{0,1\}^d \to \{0,1\}^m$ is constructed as below, with $a = \mathrm{O}\left(\log \frac{1}{\varepsilon}\right)$, $b = \mathrm{O}\left(\log n\right)$ and $d = 2b + ab \log n$, where for simplicity we assume n, a, b are integer powers of 2.*

Algorithm: RCS

Data: $\sigma_1 \leftarrow x$, *where x is an n-bit-long sample from bit-fixing source*

Result: *an m-bit-long binary string distributed ε-close to $\mathcal{U}_m$*

Initialization:

$k \leftarrow$ *lower bound on the min-entropy of the source*

$y \leftarrow \mathcal{U}_d$ *and partition* $y = (h, y_1, \ldots, y_{ab}) \in \{0,1\}^{2b} \times \{0,1,2,\ldots,n\}^{ab}$, *where h specifies a 2-universal hash function $h : \{0,1\}^b \to \{0,1\}^{mb/n}$ computable in $\mathrm{O}\left(\log n\right)$ space*

Process:

1 *generate* $\sigma_1 = x = \left(x^1, x^2, \ldots, x^{ab}\right)$ *as follows, where x^j is a copy of x for every $j \in [ab]$*

for $j = 1$ **to** $\left\lceil \log(ab) \right\rceil$ **do**

2 $\quad\sigma_2 \leftarrow (\sigma_1, \sigma_1)$

3 $\quad\sigma_1 \leftarrow \sigma_2$

end

4 $\sigma_2 \leftarrow \left(\mathsf{shift}(x^1, y_1), \ldots, \mathsf{shift}(x^{ab}, y_{ab})\right)$

5 $n' \leftarrow abn$

for $j = 1$ **to** $\left\lceil \log(a) \right\rceil$ **do**

6 $\quad\sigma_1 \leftarrow \left(\sigma_2[1], \sigma_2[2], \ldots, \sigma_2[\frac{n'}{2}]\right)$

7 $\quad\sigma_1 \leftarrow \left(\sigma_2[1] + \sigma_2[\frac{n'}{2}+1], \sigma_2[2] + \sigma_2[\frac{n'}{2}+2], \ldots, \sigma_2[\frac{n'}{2}] + \sigma_2[n']\right)$

8 $\quad n' \leftarrow \frac{n'}{2}$

9 $\quad\sigma_2 \leftarrow \sigma_1$

end

10 *partition* $\sigma_1 = (z_1, \ldots, z_n)$ *where* $|z_i| = b$ *for* $1 \leq i \leq \frac{n}{b}$

11 $\sigma_2 \leftarrow (h(z_1), \ldots, h(z_n))$

Output: σ_2

RCS uses $\mathrm{O}\left(\log(ab)\right) = \mathrm{O}\left(\log\log\frac{1}{\varepsilon} + \log\log n\right)$ passes and $\mathrm{O}\left(b + \log n\right)$ local memory. The proof of RCS as a (k, ε)-extractor for bit-fixing sources follows the same idea of Theorem 3: first assert the next-block-min-entropy $\Omega\left(\frac{kb}{n}\right)$ in step 10, then apply Lemma 7 with the random 2-universal hash function h.
